# Cytoskeleton Dependent Mobility Dynamics of FcγRIIA Facilitates Platelet Haptotaxis and Capture of Opsonized Bacteria

**DOI:** 10.3390/cells11101615

**Published:** 2022-05-11

**Authors:** Raghavendra Palankar, Laura Sachs, Jan Wesche, Andreas Greinacher

**Affiliations:** Institute for Transfusion Medicine, University Medicine Greifswald, 17489 Greifswald, Germany; laura.sachs@med.uni-greifswald.de (L.S.); jan.wesche@med.uni-greifswald.de (J.W.); andreas.greinacher@med.uni-greifswald.de (A.G.)

**Keywords:** platelets, FcγRIIA, immune complex, cytoskeleton, platelet factor 4, bacteria, micropatterns, quantum dots

## Abstract

Platelet adhesion and spreading at the sites of vascular injury is vital to hemostasis. As an integral part of the innate immune system, platelets interact with opsonized bacterial pathogens through FcγRIIA and contribute to host defense. As mechanoscavangers, platelets actively migrate and capture bacteria via cytoskeleton-rich, dynamic structures, such as filopodia and lamellipodia. However, the role of human platelet FcγRIIA in cytoskeleton-dependent interaction with opsonized bacteria is not well understood. To decipher this, we used a reductionist approach with well-defined micropatterns functionalized with immunoglobulins mimicking immune complexes at planar interfaces and bacteriamimetic microbeads. By specifically blocking of FcγRIIA and selective disruption of the platelet cytoskeleton, we show that both functional FcγRIIA and cytoskeleton are necessary for human platelet adhesion and haptotaxis. The direct link between FcγRIIA and the cytoskeleton is further explored by single-particle tracking. We then demonstrate the relevance of cytoskeleton-dependent differential mobilities of FcγRIIA on bacteria opsonized with the chemokine platelet factor 4 (PF4) and patient-derived anti-PF4/polyanion IgG. Our data suggest that efficient capture of opsonized bacteria during host-defense is governed by mobility dynamics of FcγRIIA on filopodia and lamellipodia, and the cytoskeleton plays an essential role in platelet morphodynamics at biological interfaces that display immune complexes.

## 1. Introduction

Platelets are anucleate, discoidal multifunctional cellular fragments (2–5 µm in diameter) generated from the cytoplasm of nucleated, polypoid megakaryocytes in the bone marrow and released into the blood circulation [1]. In a healthy human, peripheral blood contains about 150–400 10^9^/L platelets. Platelets rapidly adhere and aggregate at the sites of vascular injury and seal the lesions by forming hemostatic plugs. In addition, under pathophysiological conditions, undesirable activation of platelets leads to thrombosis. Platelets thus play a fundamental role in both hemostasis and thrombosis. Furthermore, as an integral part of the innate immune system, platelets cross-talk with immune cells, such as neutrophils and macrophages, by secreting proinflammatory cytokines [2]. Beyond these, platelets interact with diverse pathogens, such as viruses, bacteria, fungi, and protozoa, in a complex but delicately balanced manner, that may result in complications or in host defense [3,4,5,6]. For example, platelets interact directly and indirectly through their surface receptors with Gram-negative and Gram-positive bacteria and their secreted metabolites and proteins, leading to platelet activation [7,8,9]. However, human platelets also effectively bridge adaptive and innate immune systems to achieve host defense against bacterial pathogens [10,11,12]. These interactions and platelet responses to external biochemical and biophysical stimuli depend on specific ligand-receptor recognition, sensing, and signaling processes that result in platelet tethering, activation, adhesion, and spreading. The human platelet receptor FcγRIIA has attracted significant attention in this regard due to its relevance to fundamental platelet function in human health and disease and as a therapeutic target.

The FcγRIIA is a type 1 transmembrane glycoprotein of ≈40 kDa with a cytoplasmic region containing an immunoreceptor tyrosine-based activation motif (ITAM) with two tyrosines that undergo phosphorylation. Present at numbers ranging from 900 up to 5000 per platelet, FcγRIIA is the only FcγR expressed in human platelets, thereby making platelets the richest source of FcγRIIA in the human body [13,14]. While FcγRIIA has a low affinity for the constant region (Fc, fragment crystallizable) of monomeric IgG, immune complexes comprising IgG and IgG-opsonized targets such as bacteria are recognized with high avidity. Mechanistically, this is achieved by specific recognition of immunoglobulin (IgG) opsonized bacteria by the FcγRIIA (also called CD32a). During interaction with IgG opsonized bacteria, crosslinking of FcγRIIA triggers platelet activation. This results in extensive platelet spreading due to cytoskeletal rearrangement and subsequent directed release of alpha-granules containing bactericidal substances [15]. Beyond host defense, platelet FcγRIIA mediated platelet activation by immune complexes between anti-platelet factor 4 (PF4) IgG, and PF4 is known to induce the release of neutrophil extracellular traps (NETs) from neutrophil granulocytes in heparin-induced thrombocytopenia (HIT) [16]. More recently, platelet FcγRIIA was shown to play a vital role in inducing vaccine-induced immune thrombotic thrombocytopenia (VITT) by anti-PF4 antibodies in some individuals after vaccination with the adenoviral vector vaccines ChAdOx1 nCoV-19 (Oxford–AstraZeneca) and Ad26.COV2.S (Johnson & Johnson–Janssen) [17]. Similar to HIT, anti-PF4 antibodies have been implicated in releasing NETs from neutrophils by activated platelets in VITT patients [18]. Thus, platelet FcγRIIA plays a central role in several pathophysiological functions and interactions of platelets in the vascular system.

In platelet biology, one of the least understood phenomena is the ability of platelets to engage in the directional migration towards chemo- and hapotactic cues [19,20]. Recently, it has been shown that murine platelets exhibit cytoskeleton-dependent motility and act as mechanoscavangers of bacteria [21,22]. However, so far, the role of diffusion and mobility dynamics of FcγRIIA on human platelets during ligand sensing and its dependency on cytoskeletal integrity impacting platelet haptotaxis is not entirely clear. To assess this, we developed a reductionist approach based on micropatterning of ligands, specifically recognized by platelet FcγRIIA. Using micropatterned arrays functionalized with IgG on 2D planar surfaces and on microbeads that mimic immune complex presenting immune complexes, we demonstrate that platelet FcγRIIA mediated platelet haptotaxis is impacted by cytoskeletal integrity. In addition, taking advantage of single-particle tracking using luminescent quantum dots labeling of FcγRIIA, we further show differential receptor mobility dynamics are vital to the interaction and capture of bacteria opsonized with PF4 and anti-PF4/polyanion IgG (anti-PF4/P) from HIT patients. Our data suggest that platelets morphodynamic changes during platelet haptotaxis on immune complexes at different bio-interfaces are strongly dependent on differential mobilities of FcγRIIA and cytoskeletal integrity.

## 2. Materials and Methods

### 2.1. Reagents and Chemicals

Poly(ethylene glycol) methyl ether thiol (Mn = 800) (Cat.No. 729,108 Sigma-Aldrich (Seelze, Germany). 3-[Methoxy(polyethyleneoxy)propyl]trimethoxysilane; 90%, 6–9 PE-units (Cat.No. AB111226, ABCR GmbH & Co., Karlsruhe, Baden-Württemberg, Germany). Bovine serum albumin, Fraction V (Cat.No. BP1605-100, Fisher Scientific, Schwerte, North Rhine-Westphalia, Germany). BSA-fluorescein isothiocyanate conjugate (FITC) (Cat.No. A23015, ThermoFisher Scientific, Bremen, Germany). Water-soluble Qdot^TM^ 525 ITK^TM^ Amino (PEG) Quantum Dots (Cat. No. Q21541MP, Thermo Fisher Scientific, Darmstadt, Hesse, Germany). Monoclonal anti-human FcγRIIA Fab (Clone 7.3) and isotype control mouse IgG1 κ (MOPC31C)(Fab) from Ancell Corporation (Bayport, MN, USA). Bis[sulfosuccinimidyl] suberate (BS3), Dynabeads^®^ MyOne™ carboxylic acid, 3,3′-Dithiobis (sulfosuccinimidylpropionate) (DTSSP), (1-ethyl-3-(3-dimethylaminopropyl)carbodiimide hydrochloride) (EDC), N-hydroxysuccinimide (NHS), Fluo-4 AM cell permeant calcium (Ca^2+^) indicator, goat anti-human IgG secondary antibody conjugated to Alexa Fluor 555, goat anti-human IgG secondary antibody conjugated to FITC were purchased from Invitrogen GmbH (Darmstadt, Germany) and were used according to manufactures instructions.

### 2.2. Preparation and Functionalization of Micropatterned Arrays

Electron beam lithography (EBL) was used to prepare micropatterned arrays on glass coverslips. Briefly, coverslips were cleaned using 1:1:5 solution of ammonium hydroxide: hydrogen peroxide: deionized water at 70 °C for 10 min and dried under nitrogen stream and treated for 20 min in UV/Ozone ProCleaner Plus (BioForce Nanosciences Inc., Ames, IA, USA). Cleaned coverslips were then sputter-coated with chromium (Q150T ES, Quorum Technologies, Lewes, UK), followed by gold to create a conductive layer. To prevent nonspecific interactions beyond functionalized micropatterns, gold-coated coverslips were first functionalized with poly(ethylene glycol) methyl ether thiol (Mn = 800) (Cat.No. 729,108 Sigma-Aldrich) at a concentration of 5 mg/mL in deionized water for 24 h to form a self-assembled monolayer. To create micropatterned microarrays, 5% *v*/*v* bovine serum albumin, Fraction V (Cat.No. BP1605-100, Fisher Scientific) in deionized water was spin-coated (DELTA6 RC TT, Süss Micro Tec) for 40 s at 2000 rpm on glass coverslips coated with a self-assembled monolayer of PEG-thiol. Electron beam lithography was performed in a Zeiss Supra 40 VP scanning electron microscope (SEM) equipped with an ELPHY Quantum EBL system (Raith GmbH). Different electron beam dosages were tested starting from 10 up to 100 μC/cm^2^ at increments of 10 μC/cm^2^. The final optimal electron beam dosage of 80 μC/cm^2^ was chosen to produce high fidelity BSA micropatterns in a micropatterns of different shapes (square and circular shape with varying sizes and inter-pattern distances) were created to assess whether platelets are able to address morphodynamic changes during adhesion and spreading process specifically. The micropatterns used for functionalization were patterned within a rectangular area of 1 mm × 5 mm in dimension to minimize sample volume during adhesion assays. Electron beam exposed BSA-coated glass coverslips were developed in deionized water for 10 min, dried under a stream of nitrogen, and stored in airtight containers at 4 °C until further use. BSA-based micropatterns were used within 72 h of preparation.

### 2.3. Preparation of Heat-Aggregated Human IgG and Functionalization of Micropatterns and Microbeads

Heat-aggregated human IgG (Agg-IgG) was prepared by heating purified human IgG (Jackson ImmunoResearch Laboratories, Inc. Baltimore Pike, West Grove, PA, USA) at 5 mg/mL in PBS for 25 min at 63 °C in a water bath, followed by centrifugation at 10,000× *g* for 5 min. The supernatant was collected, stored at 4 °C, and used within 24 h of preparation. Protein concentration was determined by bicinchoninic acid (BCA) protein assay kit using BSA as standard (Sigma-Aldrich Chemie GmbH, Munich, Germany). Agg-IgG was immobilized on BSA micropatterns and carboxylic microbeads using single-step conjugation chemistry through amine-reactive N-hydroxysulfosuccinimide (sulfo-NHS) ester using homobifunctional 3,3′-DTSSP and via EDC following manufacturers protocols, respectively.

### 2.4. Preparation and Functionalization of Micropatterned Arrays with Live E. coli and Platelet Adhesion Assay

Live bacteria were immobilized on micropatterns using 1-Ethyl-3-(3-dimethylaminopropyl)-carbodiimide (EDC) and N-hydroxysuccinimide (NHS) chemistry, as previously described [23] Briefly, *E. coli* KPM121 (Δ*waaA*) grown overnight in LB media were washed thrice in PBS and were suspended in ice-cold PBS (OD of 0.8). Bacteria immobilized on micropatterns were first incubated with 20 μg mL^−1^ PF4 for 30 min at 4 °C, and unbound excess was gently rinsed in cold PBS, followed by incubation for 30 min with 10 μg mL^−1^ anti-PF4/P IgG purified from HIT patient sera. PF4 and anti-PF4/P IgG opsonized bacteria were then pipetted onto BSA micropatterns pretreated and derivatized with EDC (1 mg mL^−1^) and NHS (0.1 mg mL^−1^), and incubated for 30 min at 4 °C on a horizontal shaker. Non-adherent bacteria were removed by gently rinsing the micropatterns in cold PBS. The binding of anti-PF4/P IgG was verified by immunofluorescence using goat anti-human IgG secondary antibodies conjugated to Alexa Fluor^®^ 488. Platelet adhesion assay was performed at 37 °C for 30 min, and samples were fixed 2% *v*/*v* paraformaldehyde/PBS, pH 7.5 for 30 min, followed by rinsing quenching for 5 min in 30 mM glycine/PBS, pH 7.5.

### 2.5. Platelet Preparation, Adhesion Assays, Ca^2+^ Mobilization, and Fluorescence Microscopy

Platelets were isolated as described in [24]. All adhesion assays were performed with washed platelets in modified Tyrodes buffer supplemented with albumin, glucose, MgCl_2,_ and CaCl_2_. Glass coverslips with functionalized micropatterned arrays were mounted on the self-adhesive underside of bottomless chamber slides (sticky-Slide VI 0.4, Ibidi, Munich, Germany). For experiments evaluating Ca^2+^ transient imaging in platelets, washed platelets were incubated with 5 μM Fluo-4 AM in the dark for 30 min at room temperature. Excess Fluo-4 AM was removed by centrifuging the platelets at 750 g for 5 min. Platelet adhesion assays and fluorescence imaging of Ca^2+^ mobilization transients during platelet adhesion assays were performed by exiting Fluo-4 AM with a 488 nm laser on a Leica SP5 confocal laser-scanning microscope. For inhibition experiments, platelets (50 µL at 10,000 platelets/µL) were pretreated for 30 min at 37 °C with the cytoskeletal inhibitors cytochalasin D at 25 μM, blebbistatin at 10 μM, and the receptor blockers anti-FcγRIIA mAb (clone IV.3, purified from hybridoma) at 10 μg/mL, and abciximab at 10 μg/mL. For immunofluorescence microscopy, platelets were fixed in 2% *v*/*v* paraformaldehyde/PBS, pH 7.5, for 30 min, then the excess was rinsed in PBS and its quenching for 5 min in 30 mM glycine/PBS, pH 7.5. Actin cytoskeleton was labeled with 10 pM Phalloidin ATTO 550. Confocal fluorescence images from six different regions of interest (ROIs) were acquired for each experimental condition, with experiments performed with platelets isolated from six healthy donors to quantify platelet adhesion on micropatterns. Platelet adhesion was calculated as percent micropatterned area coverage.

### 2.6. Analysis of Platelet Filopodia Number and Length

The analysis of fixed platelets labeled for F-actin with fluorescent phalloidin was performed on ImageJ using the plugin Filopodyan [25]. Briefly, images of platelets labeled for F-actin with fluorescent phalloidin were imported into ImageJ. Image segmentation was performed using the Triangle algorithm to detect object pixels that produce a weak peak in the histogram, followed by filtering false-positive events. The final output files contain a descriptive matrix of platelet filopodia numbers and length analysis.

### 2.7. Quantification of Platelet Spread Area and Platelet Morphodynamics

Single platelet spread area on micropatterns and microbead arrays was quantified by Time-lapse. Images of platelets loaded with Fluo-4 AM spreading on micropatterns were acquired for 10–15 min at an interval of 30 s on a Leica SP5 confocal laser-scanning microscope. Image analysis was performed using image processing ADAPT -Automated Detection and Analysis of ProTrusions plugin for ImageJ [26]. Time-series images of the Fluo-4 AM channel were then imported into ImageJ running the ADAPT plugin. A Gaussian filter was applied to minimize the noise, followed by grey-level thresholding to create a binary image. The platelet membrane boundary was taken as pixels bordering segmented regions. The resulting segmentation map was used as the seed for the region-growing algorithm in the next time point of the time-series frame. A change in platelet area over time was plotted from the final output data matrix.

### 2.8. SEM Analysis

Platelets were fixed with 2.5% glutaraldehyde in PBS for 20 min, followed by post-fixation with 1% osmium tetroxide (OsO4) in PBS for 20 min. Next, platelets were dehydrated in graded aqueous ethanol solutions from 30% to 96% (each for 5 min), and then in 100% ethanol (three steps of 5 min each). Samples were dried in Polaron Critical Point Dryer (Quorum Technologies Ltd., Kent, UK) and coated with a thin layer of gold in sputter coater images were acquired in Zeiss Supra 40 VP SEM.

### 2.9. Preparation of Monovalent QD and Monoclonal anti-Human FcγRIIA Fab Conjugate

Covalent conjugation of monoclonal mouse anti-human FcγRIIA Fab fragments to QD 525 PEG-amine using the homobifunctional amine to amine crosslinker bis [sulfosuccinimidyl] suberate (BS3) was performed according to manufactures protocol. Briefly, to prepare monovalent QDs conjugated to Fab fragment (QD to Fab ratio 1:1), 100 µL of 80 nM QD 525 PEG-amine in 50 mM borate buffer, pH 8.3 were washed and transferred to PBS, pH 7.4, followed by activation in the presence of 100 µM BS3. Excess BS3 was removed by passing the activated QD through the desalting column. For conjugation, 80 nM of activated QD in PBS, pH 7.4 were incubated with 80 nM monoclonal anti-human FcγRIIA Fab fragments for 2 h at 4 °C. This was followed by incubation with 1 mg/mL BSA to block all available surface reactive groups and reduce non-specific interactions. The remaining free functional groups were quenched with 5 mM glycine, followed by centrifugal washing using ultrafiltration (100 kD). The QD-Fab conjugate was suspended in sterile modified Tyrodes buffer and stored at 4 °C until further use. As an isotype control, QD 525 PEG-amine were conjugated to mouse IgG1κ following the same procedure as above. Conjugation of QD to Fab was visualized and verified upon electrophoretic separation in 1.5% agarose gel followed by visualization in UV transilluminator (excitation at 405 nm). Characterization of colloidal parameters such as size and zeta potential were performed by dynamic light scattering (DLS) in a fixed scattering angle Zetasizer Nano-S system (Malvern Instruments Ltd., Malvern, United Kingdom). The hydrodynamic diameter (nm) was measured at 25 °C, and light scattering was detected at 173°. Surface zeta potential (ζ, mV) was performed in folded capillary zeta cells (DTS1070, Malvern Instruments Ltd., Malvern, UK). Data analysis was performed using Zetasizer software, Version 7.13 (Malvern Instruments Ltd., Malvern, UK).

### 2.10. Labelling and Imaging of FcγRIIA on Platelets with Monovalent QD Conjugated to Anti-Human FcγRIIA Fab (QD-Fab)

50 µL of washed platelets (10 × 10^4^/µL) were incubated with QD-Fab at a final concentration of 80 pM for 5 min at RT in modified Tyrodes buffer, followed by washing through centrifugation at 650× *g* for 10 min to remove unbound QD- Fab. Labeled platelets were allowed to adhere and spread in the presence of thrombin (0.005 U/mL) on fibrinogen (10 µg/mL) and blocked with BSA (1% *v*/*v*) coated glass-bottomed dishes (Ibidi, Germany) for 15 min at 37 °C. Imaging of QD-Fab bound to FcγRIIA on platelet was performed on a Leica AM TIRF system (Leica, Wetzlar, Germany). The TIRF microscope was custom equipped by the manufacturer with a 405-nm 50-mW diode laser, 100X HC PL APO CORR NA 1.47 oil immersion objective. TIRF angle of the evanescent wave was set to a penetration depth of 110 nm. Time-lapse image series were captured at a frequency of 12.8 Hz (78 ms/frame), and 1000 individual frames were acquired using Leica DFC360 FX monochrome digital charge-coupled device (CCD) camera with sensor cooling set to −20 °C. The dynamics of individual QD-Fab bound to FcγRIIA on platelets were calculated from a series of 75 images spanning a total time of 5.8 s to avoid lateral drift from the imaging hardware and minimize artifacts due to rapid and continuous changes in platelet shape changes during adhesion and spreading on fibrinogen.

### 2.11. Single-Particle Tracking and Analysis of QD-Fab on Platelet Membrane

To assess the dynamics of a single QD-Fab labeled FcγRIIA on platelets, image sequences acquired from TIRF microscopy were deconvoluted, and then corrected for drift in *x-y* axis using DeconvolutionLab2 and Manual Drift Correction plugins, respectively, on open-source image-processing package Fiji [27,28]. Visualization and rendering of trajectories from single QD-Fab bound to individual FcγRIIA were performed by single-particle tracking (SPT) on deconvoluted image series using TrackMate plugin in Fiji [29]. To calculate diffusion coefficients (*D*) from mean squared displacement (MSD) from dynamics of FcγRIIA receptor mobility, selected image series, lateral positions (*x* and *y*) from QD-Fab particle trajectories, and corresponding intensities of individual QD-Fab from TrackMate analysis were imported into SpatTrack, a MatLab based image analysis toolbox [30]. The calculated MSD was fitted using taking into consideration the combination of anomalous and directed diffusion of subpopulations FcγRIIA of moving by confined and directed transport on platelet membrane due to contribution of subcortical cytoskeletal mesh to receptor diffusion as follows:

$$\mathrm{MSD}\;\left(t\right)=4D_{\alpha \;}t^{\alpha }+v^{2\;}t^{2}$$

where *D* is the diffusion constant, *v*—velocity, *t*—time lag, and *α*—anomalous exponent.

Track Length (*L)*: QD-Fab bound to FcγRIIA on platelets was carried out using Imaris Spot Tracker package (Imaris version 7.6.5, Bitplane, Switzerland). Track length, *L*, is the total length of displacements of QD-Fab bound to FcγRIIA within the track between the measured time point indexes as follows:

$$L=\overset{t_{l}}{\underset{t-t_{f}+1}{\sum }}\sqrt{D_{x}{\left(t,t-1\right)}^{2}+D_{y}{\left(t,t-1\right)}^{2}}$$

where *L* is track length in µm, *t_l_* and *t_f_* are the first- and last-time index of track, and *D* is track displacement length of QD-Fab bound to FcγRIIA in *x* and *y* position at time index *t*.

### 2.12. Statistical Analysis

Statistical analyses were performed with GraphPad Prism version 7.03 software. Data are presented as mean ± SD and 10–90 percentile range.

## 3. Results

### 3.1. Platelet Cytoskeletal Integrity Is Indispensable for FcγRIIa Mediated Adhesion and Spreading on IgG Micropatterns

**Figure 1:**
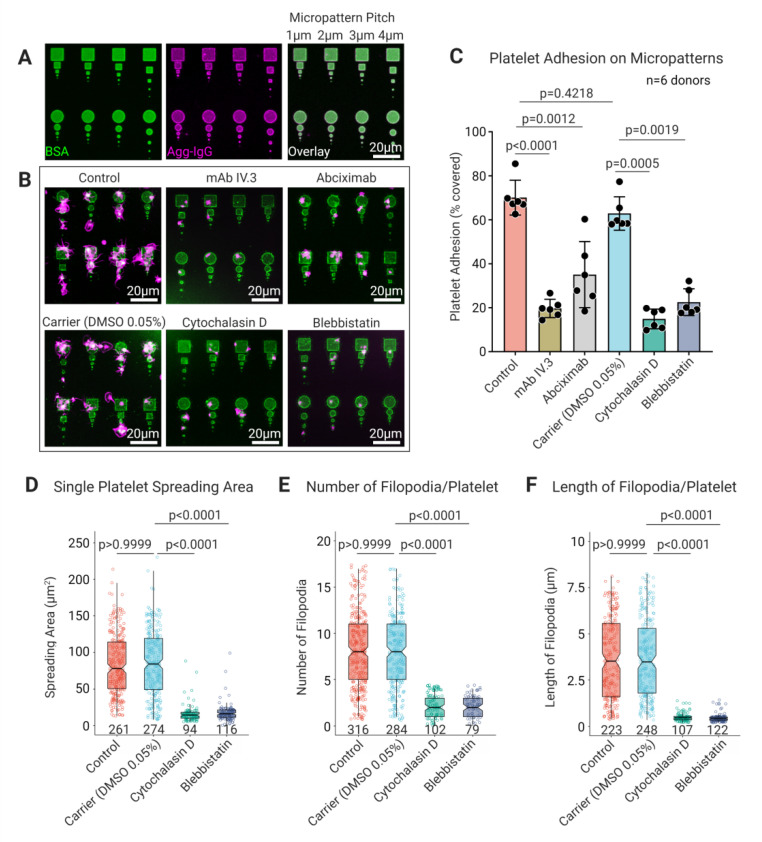
Specific recognition of Agg-IgG on micropatterns depends on platelet FcγRIIA mediated interactions, and cytoskeletal integrity is necessary for platelet adhesion and spreading. (**A**) CLSM images of micropatterned arrays of bovine serum albumin (BSA 4%) spiked with 1% BSA-FITC (in green) fabricated using electron beam lithography with different sizes inter-pattern pitch distances. Micropatterns were functionalized by covalent conjugation of heat aggregated human IgG (Agg-IgG detected using goat anti-human IgG Alexa 555 in magenta) to BSA in a one-step reaction using bifunctional linker 3,3′-Dithiobis(sulfosuccinimidylpropionate) (DTSSP); (**B**) representative confocal fluorescence microscopy images; and (**C**) analysis of the effect on platelet adhesion upon of pretreatment of platelets with FcγRIIA blocking mAb IV.3 (4 μg/mL), integrin αIIbβ3 blocking antibody, cytochalasin D (25 μM), and blebbistatin (10 μM) shows significant reduction in comparison with untreated platelets (control) and DMSO 0.05% (as carrier) on their spreading on Agg-IgG functionalized micropatterns (platelet F-actin in magenta, Agg-IgG detected using goat anti-human IgG Alexa 488 in green). Data represent mean ± SD from *n* = 6 donors, and statistical comparisons were performed using one-way ANOVA, followed by Tukey’s multiple comparisons for spreading area measurements. Inhibition of platelet F-actin polymerization by cytochalasin D (25 μM) and myosin II by ATPase inhibitor blebbistatin (10 μM) significantly reduces (**D**) platelet spreading area, (**E**) filopodia number, and (**F**) their length in single platelets in comparison with untreated platelets (control) and DMSO 0.05% (as carrier) on Agg-IgG functionalized micropatterns. Statistical comparisons were performed by one-way ANOVA on ranks (non-parametric) followed by Dunn’s multiple comparisons test. Boxplots extend from 10 to 90 percentiles, and *p* < 0.05 is significant.

To assess the role of platelet FcγRIIA and cytoskeleton on adhesion, spreading, and directional guidance of platelets, we prepared high-fidelity micropatterned arrays differing in their geometry (squares and circular discs), size (2D adhesive area), and inter-pattern distance (pitch) by electron beam lithography and functionalized them with heat aggregated IgG (Agg-IgG) (Figure 1A, Supplementary Figures S1–S3). The non-patterned surface surrounding the micropatterns was passivated with a self-assembled monolayer of polyethylene glycol, offering a non-adhesive surface. In the absence of pharmacological blockers and inhibitors of FcγRIIA, αIIbβ3, actin, and myosin IIA, platelets adhered specifically (Control 70.1% ± 7.9%), and showed extensive spreading and numerous filopodia (Figure 1B,C) on Agg-IgG micropatterns, irrespective of the micropattern shape and dimension. Blocking of FcγRIIA interaction with IgG by pretreating platelets with mAb IV.3 revealed a significant reduction in platelet adhesion on Agg-IgG micropatterns (11.61% ± 4.42%; *p* < 0.0001) compared to untreated platelets (Figure 1B,C). Additionally, specific blocking of platelet αIIbβ3 using abciximab decreased platelet adhesion on Agg.IgG micropatterns (35.08% ± 15.05%; *p* = 0.0012) (Figure 1B,C). This was expected, since integrin αIIbβ3 is known to facilitate firm adhesion and spreading of platelets during FcγRIIA mediated platelet activation on IgG passivated surfaces [31]. Next, to assess the contribution of platelet cytoskeleton in FcγRIIA mediated platelet adhesion on Agg-IgG micropatterns, platelets were pretreated with cytochalasin D and blebbistatin to inhibit F-actin assembly and interfere with actomyosin complex formation by blocking myosin-ADP-Pi, respectively. Surprisingly, we observed a reduction in platelet adhesion on Agg-IgG micropatterns upon cytochalasin D (14.91% ± 4.5%; *p* = 0.0005) and blebbistatin (22.54% ± 6.1%; *p* = 0.0019) pretreatment of platelets. Subsequent analysis of single adherent platelets revealed reduced spreading area in the presence of cytochalasin D (16.6 µm^2^ ± 12.3; *p* < 0.0001) and blebbistatin (17.7 µm^2^ ± 11.9; *p* < 0.0001) in comparison to untreated platelets (Control: 83.7 µm^2^ ± 41.5 and Carrier (DMSO): 85.2 µm^2^ ± 44.9) (Figure 1D). Next, we analyzed the number and length of filopodia on single adherent platelets on Agg-IgG micropatterns. Untreated platelets formed several (Control: 8.03 ± 4.15 and Carrier (DMSO): 7.77 ± 3.93 per single platelet) long filopodial extensions (Control: 3.78 µm ± 2.32 and Carrier (DMSO): 3.75 µm ± 2.21 per single platelet). Conversely, fewer and shorter filopodia were visible in platelets pretreated with cytochalasin D (0.38 µm ± 0.095 per single platelet; *p* < 0.0001) and blebbistatin (0.38 µm ± 0.1 per single platelet; *p* < 0.0001) (Figure 1E,F). However, it is important to note that the platelet adhesion on Agg-IgG was independent of the shape of the micropatterns.

### 3.2. Platelet Haptotaxis on IgG Planar Micropatterns Is Mediated by Dynamic Membrane Protrusions

**Figure 2:**
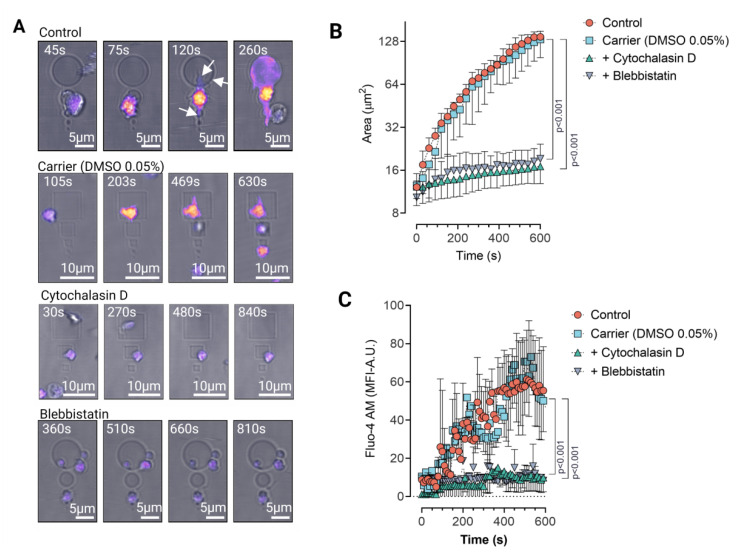
Platelets use filopodia as antennae to seek out and adhere to Agg-IgG on micropatterns through FcγRIIA and exhibit haptotaxis along laterally increasing Agg-IgG density on 2D planar micropatterns. (**A**) Platelets preloaded with Ca^2+^ indicator dye Fluo-AM were incubated on micropatterns functionalized with Agg-IgG were imaged by time-lapse video microscopy. Representative time-lapse images of Ca^2+^ release and platelet morphodynamics during adhesion and spreading on Agg-IgG micropatterns with a pitch distance of 1µm show platelet filopodia actively scan their surrounding microenvironment as platelets adhere, spread in a polarized manner resembling haptotaxis, and become activated on Agg-IgG micropatterns. This process is abrogated upon pretreatment of platelets with F-actin polymerization inhibitor cytochalasin D (25 μM) and myosin II ATPase inhibitor blebbistatin (10 μM) (see Supplementary Videos S1–S4); (**B**) Analysis of platelet morphodynamics (shape changes over time) and (**C**) changes in calcium binding dye Fluo-4 AM fluorescence (mean fluorescence intensity- MFI) during intracellular calcium release from time-lapse video microscopy reveals platelets undergo minimal morphodynamics upon pretreatment with F-actin polymerization inhibitor cytochalasin D (25 μM) and myosin II ATPase inhibitor blebbistatin (10 μM), indicating Agg-IgG sensing by platelets through their FcγRIIA is dependent on the stability of the platelet cytoskeleton, which in turn regulates filopodia formation. Data represents mean ± SD values from *n* = 6 (region of interests) from 6 donors. Fluo-4 AM calcium dynamics show mean ± SD from *n* = 3 (region of interests). Statistical comparisons were performed by one-way ANOVA on ranks (non-parametric) followed by Dunn’s multiple comparisons test. Boxplots extend from 10 to 90 percentiles, and *p* < 0.05 is significant.

Based on our observations of platelets during adhesion and spreading assays on 2D planar micropatterns, we hypothesized (i) whether platelets display active morphodynamics coupled with haptotaxis on Agg-IgG micropatterns, and (ii) how filopodia and cytoskeleton contribute to this phenomenon. Using time-lapse fluorescence video microscopy, we followed single platelet adhesion on Agg-IgG micropatterns with 1µm inter-pattern pitch distance and assessed platelet shape change and calcium release in single platelets (Figure 2A). In control platelets, soon after adhesion, we observed that platelets extend filopodia and adapt to the Agg-IgG micropattern shape while undergoing extensive membrane expansion through dynamic shape changes, and start spreading between micropatterns (Figure 2B, Supplementary Videos S1 and S2). Additionally, we observed an intermittent increase of calcium release bursts (Figure 2C) during platelet activation and morphodynamic changes on untreated platelets on Agg-IgG micropatterns, whereas, upon cytochalasin D and blebbistatin treatment, filopodia formation and platelet morphodynamics changes were abolished entirely, and platelets showed no visible increase in calcium release (Figure 2A,B, Supplementary Videos S3 and S4). Taken together, our results demonstrate that even though FcγRIIA is sufficient for the initial specific recognition of Agg-IgG for firm platelet adhesion, spreading, and subsequent FcγRIIA-mediated activation on Agg-IgG micropatterned planar surfaces, integrin αIIbβ3, and an intact platelet cytoskeleton are indispensable.

### 3.3. Platelet FcγRIIA Mediates Adhesion to ‘Bacteriamimetic’ IgG Opsonized Microbeads and Platelet Spreading Is Cytoskeleton Dependent

**Figure 3:**
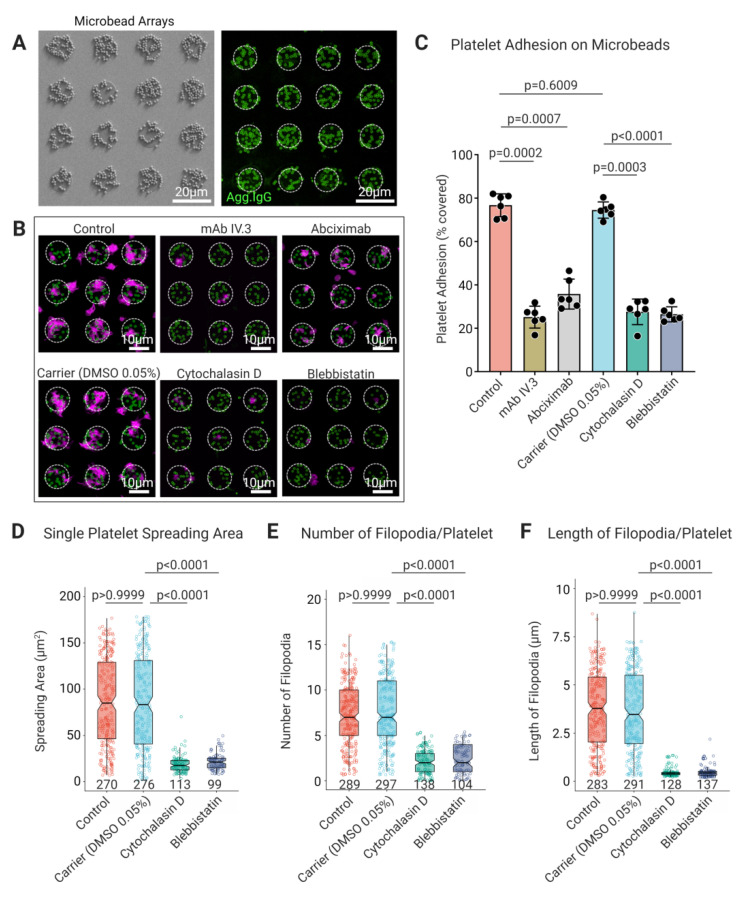
Platelet cytoskeletal destabilization leads to a decrease in platelet spreading on Agg-IgG functionalized ‘bacteriamimetic’ microbeads arrays due to abrogation of filopodia formation (**A**) SEM and CLSM images (detected using goat anti-human IgG Alexa 488 in green) of Agg-IgG functionalized ‘bacteriamimetic’ microbead arrays; (**B**) Representative CLSM images of platelet adhesion and (**C**) analysis of spreading area of single platelets pretreated with FcγRIIA blocking mAb IV.3 (4 μg/mL), integrin αIIbβ3 blocking antibody, cytochalasin D (25 μM), and blebbistatin (10 μM) shows significant reduction in comparison with untreated platelets (control) and DMSO 0.05% (as a carrier) on their spreading on Agg-IgG functionalized ‘bacteriamimetic’ microbeads arrays (platelet F-actin in magenta, Agg-IgG detected using goat anti-human IgG Alexa 488 in green). Data represent mean ± SD from *n* = 6 donors, and statistical comparisons were performed using one-way ANOVA, followed by Tukey’s multiple comparisons test. Inhibition of platelet F-actin polymerization by cytochalasin D (25 μM) and myosin II ATPase inhibitor blebbistatin (10 μM) significantly reduces (**D**) spreading area, (**E**) number, and (**F**) length of filopodia in single platelets in comparison with untreated platelets (control) and DMSO 0.05% (as carrier) on Agg-IgG functionalized ‘bacteriamimetic’ microbeads arrays. Statistical comparisons were performed by one-way ANOVA on ranks (non-parametric) followed by Dunn’s multiple comparisons test. Boxplots extend from 10 to 90 percentiles, and *p* < 0.05 is significant.

Based on our observations of platelet interactions on 2D planar surfaces immobilized with Agg-IgG, we next hypothesized whether platelets behave similarly on complex geometries that resemble IgG opsonized bacteria. It has been well established that platelets interact with IgG opsonized bacteria primarily through FcγRIIA. However, these interactions are augmented by complex and multiple direct and indirect recognition between several platelet receptors and bacterial surface components [32]. To avoid the secondary interactions that may lead to measurement artifacts and compromise our understanding of the underlying mechanisms of host-pathogen interactions, synthetic microbeads decorated with bacterial proteins mimicking bacterial surfaces are currently being used [7,8]. Within this context, to dissect the role of platelet FcγRIIA mediated interaction with opsonized bacteria, we immobilized ‘bacteriamimetic’ microbeads covalently coated with Agg-IgG on micropatterned arrays (Figure 3A). Incubation with platelets showed that platelets adhered (Control 76.73% ± 5.2%) to Agg-IgG coated bacteriamimetic microbeads on micropatterns (Figure 3B,C). Reduced platelet adhesion was observed upon the blocking of FcγRIIA (25.14% ± 5.05%; *p* = 0.0002) and αIIbβ3 (35.78% ± 6.9%; *p* = 0.0007) (Figure 3B,C). Similarly, platelet adhesion to Agg-IgG bacteriamimetic microbeads was significantly reduced in platelets pretreated with cytochalasin D (25.3% ± 1.77%; *p* = 0.0003) and blebbistatin (27.6% ± 5.9%; *p* < 0.0001) (Figure 3B,C). Further assessment of morphological parameters of platelets pretreated with cytochalasin D and blebbistatin showed a significant reduction of single platelet spread area, number, and length of filopodia compared with untreated and carrier control platelets (Figure 3D–F). Specifically, a decreased single platelet spreading area was observed in the presence of cytochalasin D (18.9 µm^2^ ± 8.9; *p* < 0.0001) and blebbistatin (21.7 µm^2^ ± 9; *p* < 0.0001) in comparison with untreated platelets (85.6 µm^2^ ± 46.9) and carrier (DMSO) (86.1 µm^2^ ± 51). An analysis of number and length of filopodia on single adherent platelets on Agg-IgG coated bacteriamimetic microbeads revealed untreated platelets formed several (Control: 7.63 ± 4.22 and Carrier (DMSO): 7.61 ± 4.12 per single platelet) and longer filopodial protrusions (Control: 3.85 µm ± 2.08 and Carrier (DMSO): 4.33 µm ±2.12 per single platelet). Conversely, fewer and shorter filopodia were visible in platelets pretreated with cytochalasin D (2.19 ± 1.4; *p* < 0.0001 and 0.26 µm ± 0.1; *p* < 0.0001, respectively per single platelet) and blebbistatin (3.44 ± 2.1; *p* < 0.0001 and 0.3 µm ± 0.08; *p* < 0.0001, respectively per single platelet).

**Figure 4:**
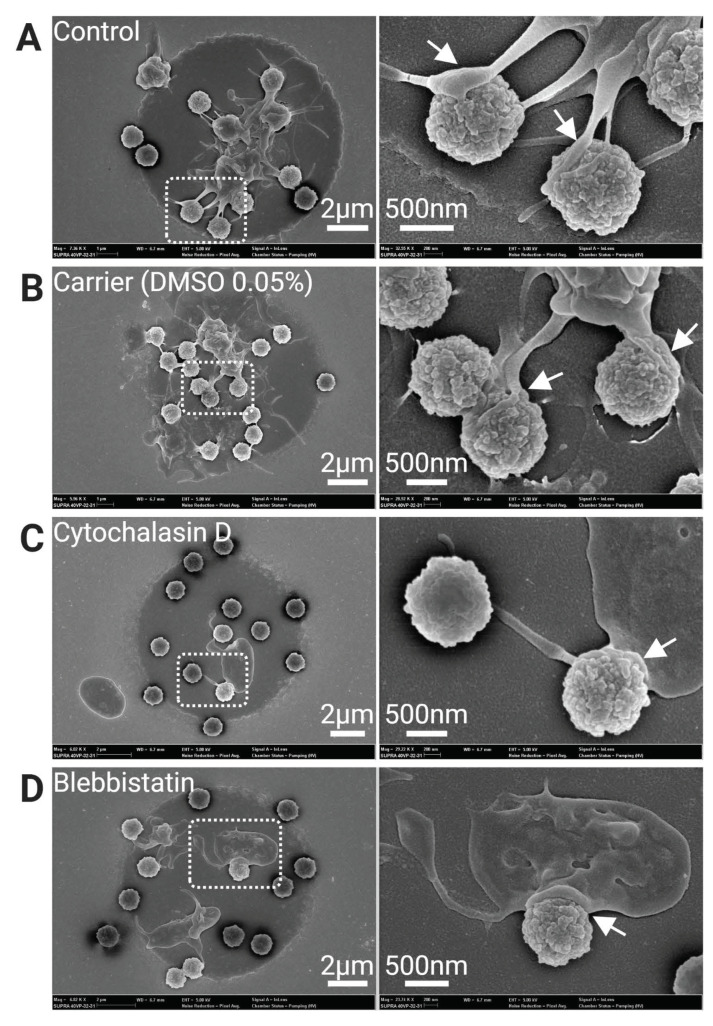
Scanning electron microscopy (SEM) of platelets interaction with Agg-IgG coated bacteriamimetic microbeads on micropattern. In controls (**A**,**B**), platelets can be seen adhering and spreading on the Agg-IgG beads. Extensive filopodia and membranous lamellipodia (indicated arrowheads) are visible in control platelets but not in platelets treated with cytoskeletal inhibitors Cytochalasin D and Blebbistatin (**C**,**D**).

Surprisingly, in contrast to the platelet haptotaxis observed on planar 2D ligand gradient micropatterns (Figure 2A), haptotactic migration was absent on Agg-IgG bacteriamimetic microbeads. Instead, analysis of platelet adhesion phenotype by SEM showed filopodial extensions tightly adhering and wrapping around the Agg-IgG coated microbeads, but not in platelets treated with cytoskeletal inhibitors (Figure 4).

### 3.4. Lateral Mobility of FcγRIIA Is Dependent on Cytoskeletal Integrity

**Figure 5:**
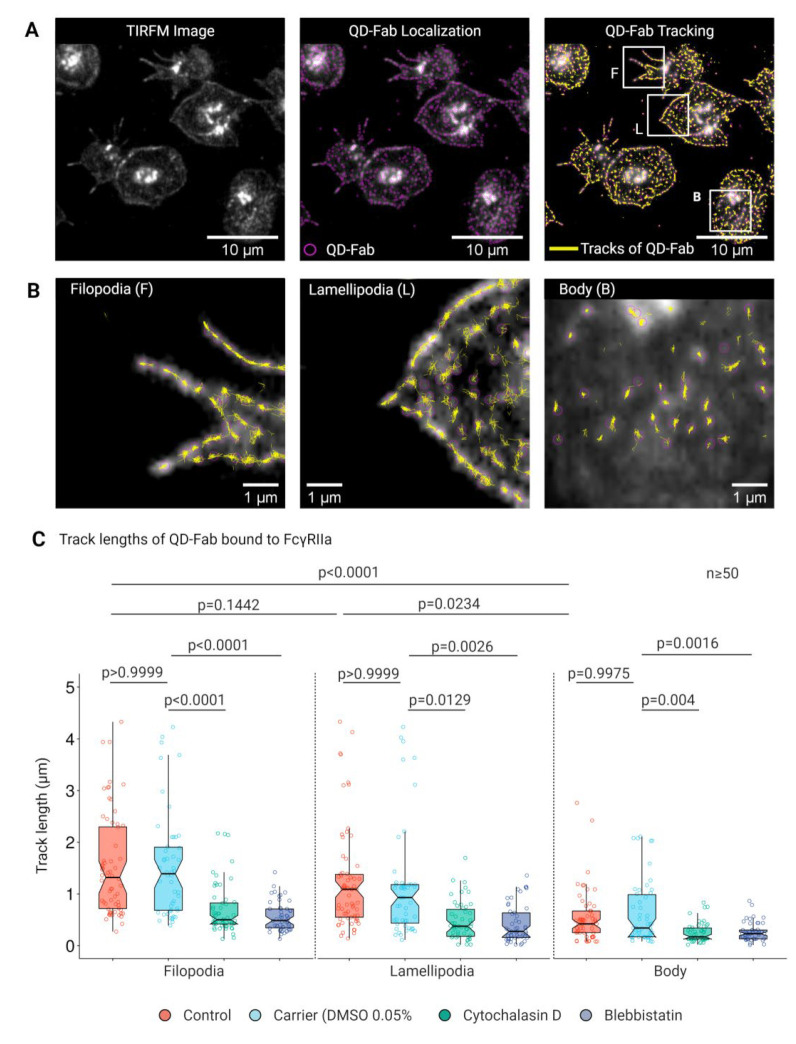
Single-particle tracking of FcγRIIA on platelets using QD-Fab. (**A**) Fluorescence micrographs showing platelets labeled with QD + anti-FcγRIIA Fab bound to FcγRIIA followed by localization of single FcγRIIA by detection of single QD fluorescence emission (magenta) and overlay of tracks (yellow) identified from (**B**) time-lapse images of single QD-Fab bound to individual FcγRIIA along the filopodia (F), lamellipodia (L), and platelet body; Analysis of track length (**C**) from trajectories of single QD-Fab bound to FcγRIIA on filopodia, lamellipodia, and platelet body. Statistical comparisons were performed by one-way ANOVA on ranks (non-parametric), followed by Dunn’s multiple comparisons test. Boxplots extend from 10 to 90 percentiles, and *p* < 0.05 is significant and *n* ≥ 50 single QD-fab per dataset.

Since platelet cytoskeletal integrity plays a crucial role in filopodia and lamellipodia mediated adhesion, spreading and haptotaxis through FcγRIIA on 2D planar surfaces and microbead arrays functionalized with Agg-IgG, we next asked the question as to whether platelet cytoskeleton also influences the underlying physical factor, such as mobility of FcγRIIA. We used QDs for single-particle tracking (SPT) of FcγRIIA on the platelet plasma membrane to assess this. Specific labeling, detection, and monitoring of single FcγRIIA in live platelets spread on fibrinogen surface were carried out using monovalent QD conjugated to a single monoclonal Fab fragment (QD-Fab) against the exofacial epitope of FcγRIIA that recognizes the Fc portion of IgG (Figure 5A, Supplementary Figure S5). Assessment of single FcγRIIA track length based on SPT revealed FcγRIIA moved longer distances on filopodia (1.13 µm ± 1.12) and lamellipodia (0.80 µm ± 1.09), compared to those on the platelet body (0.48 µm ± 0.59) (Figure 5B,C, Supplementary Figure S6 and Videos S5–S7). Disruption of F-actin assembly using cytochalasin D and blebbistatin markedly decreased the track length of single FcγRIIA mobilities on platelet filopodia, lamellipodia, and body (Figure 5C).

**Figure 6:**
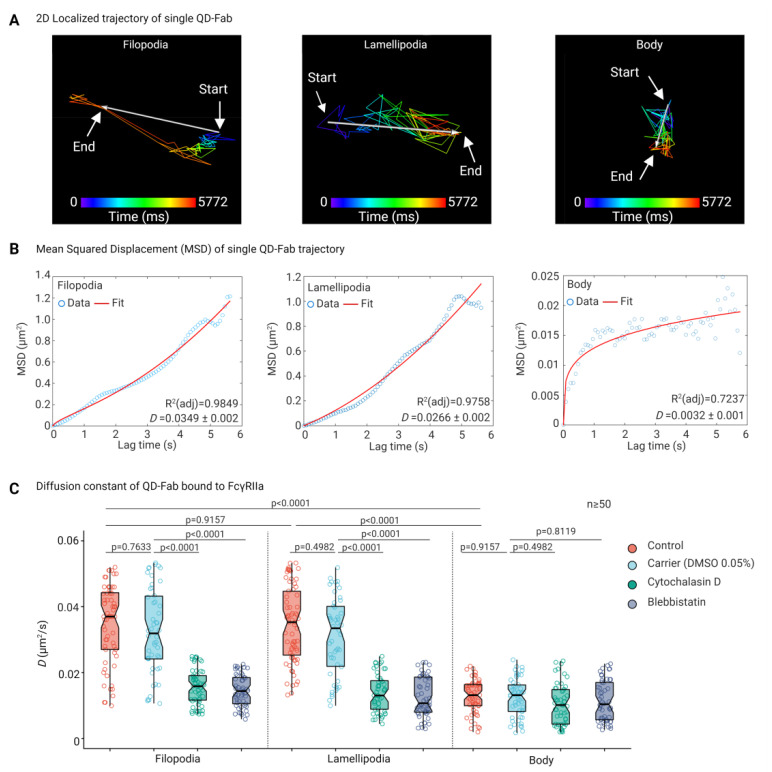
Differential lateral mobility dynamics of single FcγRIIA on platelets. (**A**) 2D localized trajectory and displacement (arrows with start and end positions) of single QD-Fab bound to FcγRIIA on platelet filopodia, lamellipodia, and platelet body as a function of lag time shown by color-coded time scale. (**B**) Mean squared displacement (MSD, µm^2^) as a function of the lag time of single QD-Fab trajectory shows the directional motion of FcγRIIA on platelet filopodia and lamellipodia and a confined motion on the platelet body (please note the different scales of the *Y*-axis in the different panels). (**C**) Comparison of the diffusion constant (*D*, µm^2^/s) of individual FcγRIIA calculated from single QD-Fab trajectory in untreated platelets (control) and DMSO 0.05% (as carrier) and after inhibition of platelet F-actin polymerization by cytochalasin D (25 μM) and myosin II ATPase inhibitor blebbistatin (10 μM). Statistical comparisons were performed by one-way ANOVA on ranks (non-parametric), followed by Dunn’s multiple comparisons test. Boxplots extend from 10 to 90 percentiles, and *p* < 0.05 is significant and *n* ≥ 50 single QD-fab per dataset.

Next, we assessed the lateral mobility dynamics of single FcγRIIA on platelets. On platelet filopodia and lamellipodia, a single QD-Fab bound to FcγRIIA showed directional motion along the axis of the filopodia and towards the edge of the cytoplasmic rim in case of lamellipodia, while on the platelet body, the motion was largely confined (Figure 6A,B and Supplementary Figure S6). Diffusion constants (*D*, µm^2^/s) of single FcγRIIA were significantly higher on platelet filopodia (0.035 µm^2^/s ± 0.012; *p* < 0.0001) and lamellipodia (0.034 µm^2^/s ± 0.011; *p* < 0.0001) compared to the platelet body (0.013 µm^2^/s ± 0.005) (Figure 6C). Disruption of platelet F-actin polymerization cytochalasin D and blebbistatin resulted in marked decrease in the FcγRIIA diffusion constant both on filopodia (0.016 µm^2^/s ± 0.005 and 0.014 µm^2^/s ± 0.005, respectively) and lamellipodia (0.014 µm^2^/s ± 0.005 and 0012 µm^2^/s ± 0.006), compared to the carrier (0.033 µm^2^/s ± 0.013 and 0.031 µm^2^/s ± 0.012). On the contrary, the FcγRIIA diffusion constant on the platelet body remained largely unchanged in the presence of cytochalasin D (0.01 µm^2^/s ± 0.006) and blebbistatin (0.011 µm^2^/s ± 0.006) compared to the carrier (0.012 µm^2^/s ± 0.006).

### 3.5. Longer Track Lengths and Higher Lateral Mobility of FcγRIIA on Platelet Filopodia and Lamellipodia Facilitate Sensing and Capture IgG Opsonized Bacterial Pathogens by Platelets

**Figure 7:**
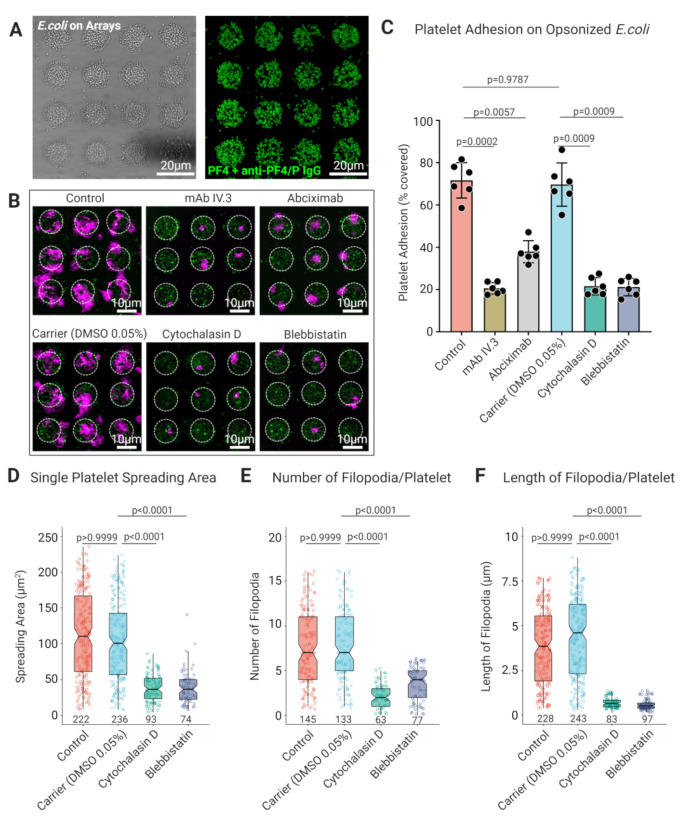
Platelets specifically recognize opsonized by PF4 + anti-PF4/P IgG via FcγRIIA using filopodial protrusions, while their adhesion and spreading are regulated by cytoskeletal rearrangement. (**A**) Light transmission and CLSM immunofluorescence micrographs of live *E. coli* KPM121 (Δ*waaA*) opsonized with PF4 + anti-PF4/P IgG immobilized on micropattern arrays (detected using goat anti-human IgG Alexa 488 in green); (**B**) Representative CLSM images of platelet adhesion and (**C**) analysis of spreading area of single platelets pretreated with FcγRIIA blocking mAb IV.3 (4 μg/mL), integrin αIIbβ3 blocking antibody, cytochalasin D (25 μM), and blebbistatin (10 μM) shows significant reduction comparison with untreated platelets (control) and DMSO 0.05% (as carrier) on their spreading on *E. coli* KPM121 (Δ*waaA*) opsonized with PF4 and anti-PF4/P IgG (platelet F-actin in magenta, patient-derived anti-PF4/P IgG detected using goat anti-human IgG Alexa 488 in green). Data represent mean ± SD from *n* = 6 donors, and statistical comparisons were performed using one-way ANOVA, followed by Tukey’s multiple comparisons test. Inhibition of platelet F-actin polymerization by cytochalasin D (25 μM) and myosin II ATPase inhibitor blebbistatin (10 μM) significantly reduces (**D**) spreading area, (**E**) number and (**F**) length of filopodia in single platelets in comparison with untreated platelets (control) and DMSO 0.05% (as carrier) on opsonized *E. coli*. Statistical comparisons were performed by one-way ANOVA on ranks (non-parametric), followed by Dunn’s multiple comparisons test. Boxplots extend from 10 to 90 percentiles, and *p* < 0.05 is significant.

To assess the biological significance of FcγRIIA mobility on platelet-bacteria interactions, we incubated platelets on micropatterns functionalized with living *E. coli* opsonized with PF4 and anti-PF4/*p* IgG (Figure 7A). A higher percentage of untreated platelets specifically adhered to opsonized *E. coli* on micropatterns (Control 74.44% ± 8.8%), whereas platelet adhesion was significantly inhibited upon blocking of FcγRIIA by antibody IV.3 (19.57% ± 3.23%; *p* = 0.0002), as well as by blocking of αIIbβ3 by abciximab (41.53% ± 5.78%; *p* = 0.0057) (Figure 7B,C). Additionally, a markedly reduced platelet adhesion to PF4 and anti-PF4/P IgG opsonized *E. coli* was observed in the presence of cytochalasin D (21.62% ± 4.11%; *p* = 0.0009) and blebbistatin (18.56% ± 2.97%; *p* = 0.0009) in comparison to the carrier (DMSO) (72.45% ± 7.9) (Figure 7B,C). Analysis of the platelet morphology of adherent platelets on opsonized *E. coli* revealed decreased single platelet spreading areas in the presence of cytochalasin D (7.72 µm^2^ ± 3.8) and blebbistatin (7.77 µm^2^ ± 4.6), in comparison with the untreated platelets (23.62 µm^2^ ± 13.4) and the carrier (DMSO) (20.1 µm^2^ ± 11.37) (Figure 7D). Furthermore, we found a reduction in the number and length of filopodial protrusions in the presence of cytochalasin D and blebbistatin (Figure 7E,F). These results are consistent with our observations of platelet adhesion, spreading, and morphometric features of filopodia observed on 2D planar and bacteriamimetic microbead micropatterns functionalized with the Agg-IgG that functions as a potent ligand for FcγRIIA.

## 4. Discussion

In human health and disease, platelet FcγRIIA plays a vital role in the pathophysiology of diseases, such as HIT and VITT, where crosslinking of platelet FcγRIIA by anti-platelet IgG antibodies occurs leading to platelet clearance, it may potentially also contribute to some forms of immune thrombocytopenia (ITP) [18,33]. Additionally, platelet FcγRIIA is also implicated in the clinical manifestation of the prothrombotic adverse drug effect HIT, leading to severe morbidity and mortality [24]. Briefly, platelet factor 4 (PF4) interacts with polyanions (P), such as the anticoagulant heparin, and undergoes conformational changes triggering an immune response. Subsequently, immune complexes formed between PF4, P, and anti-PF4/P IgG produced by the human host induce crosslinking of the FcγRIIA on platelets leading to platelet activation and generation of excess thrombin leading to thrombocytopenic symptoms. By serendipity, anti-PF4/P IgG can effectively opsonize PF4 bound to both Gram-positive and Gram-negative bacteria, triggering platelet activation and aggregation [34,35,36]. Similarly, PF4 has been shown to interact with adenoviral vector derived from chimpanzee adenovirus Y25 (ChAdOx1) and its components used for vaccination against COVID-19 [18,37,38]. Intriguingly, individuals who develop VITT post-ChAdOx1 vaccination also develop anti-PF4 antibodies, which do not depend on heparin [39].

We have proposed that the anti-PF4 immune response is an evolutionary old immune reaction bridging innate and adapted immunity allowing granulocytes and platelets to recognize various pathogens coated with PF4 and opsonized by anti-PF4/P IgG via the FcγRIIa [15,35,40]. However, the role of platelet FcγRIIA dynamics and contribution of the platelet cytoskeleton during the processes leading to firm adhesion, spreading, and platelet haptotaxis at interfaces, such as opsonized pathogens displaying the IgG as a primary ligand, have not been explored. In particular, it is nearly impossible to dissect the effects of specific pathogen-derived or platelet components in these complex processes of pathogen-platelet interactions. We overcame this inherent limitation by using minimalistic models of biological interfaces mimicking inflammatory and pathogenic circumstances using lithography and biofunctionalization techniques.

Cell protrusions are essential for motility during chemotaxis and haptotaxis [41]. During haptotaxis (directed movement), migrating cells rely on filopodial and lamellipodial protrusions that act as sensors or antennae to probe the extracellular biochemical and micro-and nano-environmental cues [42,43,44]. In platelets, upon stimulation by agonists, highly dynamic actin-rich filopodial protrusions and lamellipodia are formed [45]. Platelet filopodia are involved in sensing topographic cues to initiate platelet spreading and play a key role in hemostasis [46,47]. Previously, geometrically defined micropatterns have been found to be helpful in investigating single platelet adhesion and platelet function on various adhesive substrates [7,8,48,49,50]. We, therefore, used a systematic biomimetic approach with stepwise increasing complexity starting from planar micropatterned arrays and bacteria-like microbead-based arrays, both functionalized with Agg-IgG as a specific ligand for FcγRIIA ligand. On Agg-IgG micropatterns, we found platelet adhesion and spreading to be largely dependent on the specific interaction of FcγRIIA with its ligand and on cytoskeletal integrity.

Interestingly, we also found blocking of αIIbβ3 reduces platelet adhesion to Agg-IgG. These data agree with previous observations, which demonstrated not only FcγRIIA but also fully functional αIIbβ3 is necessary for optimal platelet adhesion on interfaces exposing IgG [51]. In addition, our data of platelet morphodynamics showed directional haptotaxis of platelets mediated by highly dynamic filopodia and lamellipodia on Agg-IgG micropatterns which was strongly abrogated upon cytoskeletal disruption. Similarly, platelets exhibited a strong dependency on FcγRIIA, and cytoskeletal integrity for adhesion on Agg-IgG coated bacteriamimetic microbeads. Surprisingly, we did not observe the directional movement of platelets on Agg-IgG microbeads. However, extensive filopodia and lamellipodia could be seen tightly interacting with the microbeads. This was, again, abrogated upon cytoskeletal disruption. One possible explanation could be that the surface topography and geometric constraints and the spatial distribution of Agg-IgG on microbeads may limit efficient platelet haptotaxis [52,53]. Another potential explanation is that platelet differentially behave depending on the three-dimensional structure of the ligand presented. On planar 2D surfaces, they primarily spread, as this is probably most relevant for wound closure in hemostasis. 3D areas, however, signal pathogens, and this induces a function of platelets related to host defense by immobilizing the pathogen and presenting it to phagocytosing cells [21]. Additionally, a predominantly localized adhesion with a lack of increased spreading compared to 2D planar micropatterns can be attributed to the self-deposition of receptor-interacting ligands at the site of adhesion [54].

To further understand the interaction of platelet FcγRIIA and the cytoskeleton in ligand recognition and haptotaxis, we investigated the lateral mobility dynamics of individual FcγRIIA. This was achieved by specific binding of an anti-FcγRIIA Fab conjugated to a quantum dot (QD). QDs are luminescent semiconductor nanoparticles with high, uniform, and photo-stable brightness, thus permitting detection to the single nanoparticle level and reliable quantification of receptor mobilities [55,56]. Our data from single-particle tracking of FcγRIIA on platelets show differential mobility of the receptor on different parts of the platelets during their morphodynamic spreading. On filopodia and lamellipodia, FcγRIIA exhibits longer track lengths and a higher diffusion constant, but not on the platelet body, which strongly depends on the cytoskeletal integrity. These data suggest a continuous actin-based “treadmilling” mechanism controls the dynamics of FcγRIIA lateral mobility on the platelets in regions where actin polymerization occurs at increased rates [57,58,59]. This, in turn, may facilitate rapid ligand engagement by FcγRIIA on filopodia and lamellipodia [60]. However, the confined mobility of FcγRIIA can potentially be attributed to diffusion barriers resulting from highly crosslinked actin and the abundance of other surface receptors within nano- and microclusters [61,62,63]. Additionally, lipid rafts may contribute to the localization and lateral mobility of FcγRIIA [64].

To explore the significance of our findings on differential mobility dynamics of FcγRIIA, we recapitulated platelet-pathogen interactions on micropatterns. To demonstrate this, we used a well-understood opsonized bacterial system involving PF4 and HIT patient-derived anti-PF4/P IgG [15,35]. Similar to the interaction of platelets with Agg-IgG coated opsonized bacteriamimetic microbeads, we observed a strong dependency on functional FcγRIIA and cytoskeletal integrity for platelet adhesion and spreading. Platelets exhibited extensive filopodia and lamellipodia, indicating that these dynamic structures are primarily used by platelets to sense opsonized bacteria. We believe such interactions may have a wide range of implications for the response of platelets to opsonized bacteria during systemic infections and during platelet-platelet interaction with IgG coated autologous cells, e.g., in autoimmune disorders such as anti-phospholipid syndrome.

Although our results show a strong dependency on human platelet FcγRIIA and cytoskeleton as key partners during platelet adhesion, spreading, and haptotaxis on biological interfaces with immune complexes, there are some limitations to our approach. In the current work, we have limited ourselves to a single ligand IgG (e.g., Agg-IgG and anti-PF4/P IgG). Thus, one cannot exclude the role of various biochemical and biophysical stimuli that platelets encounter during their interaction with IgG opsonized cells and pathogens in a vascular system. Additionally, as mouse platelets do not express an Fc-receptor, our data indicate that in vivo mouse experiments may not reflect the role of platelets in situations in which antibodies are involved. However, the modular bottom-up system of micropatterns provided here can be used to sequentially incorporate other variables to capture the complexity of platelet receptors and ligand interactions reflecting biological interfaces.

## 5. Conclusions

In summary, we demonstrate that platelet interaction via FcγRIIA at interfaces presenting IgG in close proximities (e.g., aggregated IgG or anti-PF4 IgG on opsonized pathogens) primarily occurs through cytoskeleton rich dynamic filopodial and lamellipodial extensions. In addition, single-particle tracking revealed differential mobility dynamics of human platelet FcγRIIA. Our data suggest both of these processes are mainly cytoskeleton dependent. Furthermore, using facile nano/microfabricated biomimetic minimal interfaces in combination with specific blockers and inhibitors, we show that complex interactions of platelets with molecular ligands on different geometries, including whole bacteria, can be deciphered. Such an approach can be extended to interrogate a variety of platelet receptor-mediated interactions with the potential for applications in basic biology, such as assessment of platelet function defects related to granule secretion, cytoskeleton, receptor signaling, and for drug screening.

## Data Availability

The datasets generated and analyzed during the study are available from the corresponding author.

## Supplementary Materials

The following supporting information can be downloaded at: https://www.mdpi.com/article/10.3390/cells11101615/s1, Figure S1: Schematics of micropatterned array functionalized with Agg-IgG used for platelet adhesion and spreading assays; Figure S2: Optimization of electron beam dosage for preparation of high-fidelity micropatterns; Figure S3: Line profiles along the fluorescent micropatterns; Figure S4: Characterization of quantum dots (QD) by dynamic light scattering (DLS); Figure S5: Confocal fluorescence microscopy of specific labeling of FcγRIIA on platelets by QD-Fab; Figure S6: Representative images of individual tracks and trajectories of QD-Fab bound to platelet FcγRIIA; Video S1: Control platelet adhesion and haptotaxis on Agg.IgG micropatterns; Video S2: Carrier Control (DMSO 0.05%) showing platelet adhesion and haptotaxis on Agg.IgG micropatterns; Video S3: Cytochalasin D treated platelets fail to adhere firmly and do not spread on Agg.IgG micropatterns; Video S4: Blebbistatin treated platelets adhere weakly and do not spread on Agg.IgG micropatterns; Video S5: Tracking dynamics of FcγRIIA via QD-Fab on platelet filopodia. Legends-magenta circles indicate the localization of individual QD luminescence, and yellow lines represent their computed tracks. Image interval of 78 ms, and playback is at 5 fps; Video S6: Tracking dynamics of FcγRIIA via QD-Fab on platelet lamellipodia. Legends-magenta circles indicate the localization of individual QD luminescence, and yellow lines represent their computed tracks. Image interval of 78 ms, and playback is at 5 fps; Video S7: Tracking dynamics of FcγRIIA via QD-Fab on platelet body. Legends-magenta circles indicate the localization of individual QD luminescence, and yellow lines represent their computed tracks. Image interval of 78 ms, and playback is at 5 fps.

## References

[1] Machlus K.R., Italiano J.E. (2013). The incredible journey: From megakaryocyte development to platelet formation. J. Cell Biol..

[2] Deppermann C., Kubes P. (2018). Start a fire, kill the bug: The role of platelets in inflammation and infection. Innate Immun..

[3] Ali R.A., Wuescher L.M., Dona K.R., Worth R.G. (2017). Platelets Mediate Host Defense against Staphylococcus aureus through Direct Bactericidal Activity and by Enhancing Macrophage Activities. J. Immunol..

[4] Kho S., Barber B.E., Johar E., Andries B., Poespoprodjo J.R., Kenangalem E., Piera K.A., Ehmann A., Price R.N., William T. (2018). Platelets kill circulating parasites of all major Plasmodium species in human malaria. Blood.

[5] Youssefian T., Drouin A., Masse J.M., Guichard J., Cramer E.M. (2002). Host defense role of platelets: Engulfment of HIV and Staphylococcus aureus occurs in a specific subcellular compartment and is enhanced by platelet activation. Blood.

[6] Beaulieu L.M., Clancy L., Tanriverdi K., Benjamin E.J., Kramer C.D., Weinberg E.O., He X., Mekasha S., Mick E., Ingalls R.R. (2015). Specific Inflammatory Stimuli Lead to Distinct Platelet Responses in Mice and Humans. PLoS ONE.

[7] Palankar R., Binsker U., Haracska B., Wesche J., Greinacher A., Hammerschmidt S. (2018). Interaction between the Staphylococcus aureus extracellular adherence protein Eap and its subdomains with platelets. Int. J. Med. Microbiol..

[8] Binsker U., Palankar R., Wesche J., Kohler T.P., Prucha J., Burchhardt G., Rohde M., Schmidt F., Broker B.M., Mamat U. (2018). Secreted Immunomodulatory Proteins of Staphylococcus aureus Activate Platelets and Induce Platelet Aggregation. Thromb. Haemost..

[9] Zhu W., Gregory J.C., Org E., Buffa J.A., Gupta N., Wang Z., Li L., Fu X., Wu Y., Mehrabian M. (2016). Gut Microbial Metabolite TMAO Enhances Platelet Hyperreactivity and Thrombosis Risk. Cell.

[10] Riaz A.H., Tasma B.E., Woodman M.E., Wooten R.M., Worth R.G. (2012). Human platelets efficiently kill IgG-opsonized *E. coli*. FEMS Immunol. Med. Microbiol..

[11] Stocker T.J., Ishikawa-Ankerhold H., Massberg S., Schulz C. (2017). Small but mighty: Platelets as central effectors of host defense. Thromb. Haemost..

[12] Nishat S., Wuescher L.M., Worth R.G. (2018). Platelets enhance dendritic cell responses against *S. aureus* through CD40-CD40L interactions. Infect. Immun..

[13] Arman M., Krauel K. (2015). Human platelet IgG Fc receptor FcgammaRIIA in immunity and thrombosis. J. Thromb. Haemost..

[14] Qiao J., Al-Tamimi M., Baker R.I., Andrews R.K., Gardiner E.E. (2015). The platelet Fc receptor, FcgammaRIIa. Immunol. Rev..

[15] Palankar R., Kohler T.P., Krauel K., Wesche J., Hammerschmidt S., Greinacher A. (2018). Platelets kill bacteria by bridging innate and adaptive immunity via platelet factor 4 and FcgammaRIIA. J. Thromb. Haemost..

[16] Perdomo J., Leung H.H.L., Ahmadi Z., Yan F., Chong J.J.H., Passam F.H., Chong B.H. (2019). Neutrophil activation and NETosis are the major drivers of thrombosis in heparin-induced thrombocytopenia. Nat. Commun..

[17] Greinacher A., Thiele T., Warkentin T.E., Weisser K., Kyrle P.A., Eichinger S. (2021). Thrombotic Thrombocytopenia after ChAdOx1 nCov-19 Vaccination. N. Engl. J. Med..

[18] Greinacher A., Selleng K., Palankar R., Wesche J., Handtke S., Wolff M., Aurich K., Lalk M., Methling K., Volker U. (2021). Insights in ChAdOx1 nCoV-19 vaccine-induced immune thrombotic thrombocytopenia. Blood.

[19] Lowenhaupt R.W., Miller M.A., Glueck H.I. (1973). Platelet migration and chemotaxis demonstrated in vitro. Thromb. Res..

[20] Pitchford S.C., Momi S., Baglioni S., Casali L., Giannini S., Rossi R., Page C.P., Gresele P. (2008). Allergen induces the migration of platelets to lung tissue in allergic asthma. Am. J. Respir. Crit. Care Med..

[21] Gaertner F., Ahmad Z., Rosenberger G., Fan S., Nicolai L., Busch B., Yavuz G., Luckner M., Ishikawa-Ankerhold H., Hennel R. (2017). Migrating Platelets Are Mechano-scavengers that Collect and Bundle Bacteria. Cell.

[22] Nicolai L., Schiefelbein K., Lipsky S., Leunig A., Hoffknecht M., Pekayvaz K., Raude B., Marx C., Ehrlich A., Pircher J. (2020). Vascular surveillance by haptotactic blood platelets in inflammation and infection. Nat. Commun..

[23] Louise Meyer R., Zhou X., Tang L., Arpanaei A., Kingshott P., Besenbacher F. (2010). Immobilisation of living bacteria for AFM imaging under physiological conditions. Ultramicroscopy.

[24] Greinacher A., Warkentin T.E. (2013). Heparin-Induced Thrombocytopenia. Practical Transfusion Medicine.

[25] Urbancic V., Butler R., Richier B., Peter M., Mason J., Livesey F.J., Holt C.E., Gallop J.L. (2017). Filopodyan: An open-source pipeline for the analysis of filopodia. J. Cell Biol..

[26] Barry D.J., Durkin C.H., Abella J.V., Way M. (2015). Open source software for quantification of cell migration, protrusions, and fluorescence intensities. J. Cell Biol..

[27] Sage D., Donati L., Soulez F., Fortun D., Schmit G., Seitz A., Guiet R., Vonesch C., Unser M. (2017). DeconvolutionLab2: An open-source software for deconvolution microscopy. Methods.

[28] Schindelin J., Arganda-Carreras I., Frise E., Kaynig V., Longair M., Pietzsch T., Preibisch S., Rueden C., Saalfeld S., Schmid B. (2012). Fiji: An open-source platform for biological-image analysis. Nat. Methods.

[29] Tinevez J.Y., Perry N., Schindelin J., Hoopes G.M., Reynolds G.D., Laplantine E., Bednarek S.Y., Shorte S.L., Eliceiri K.W. (2017). TrackMate: An open and extensible platform for single-particle tracking. Methods.

[30] Lund F.W., Jensen M.L., Christensen T., Nielsen G.K., Heegaard C.W., Wustner D. (2014). SpatTrack: An imaging toolbox for analysis of vesicle motility and distribution in living cells. Traffic.

[31] Zhi H., Dai J., Liu J., Zhu J., Newman D.K., Gao C., Newman P.J. (2015). Platelet Activation and Thrombus Formation over IgG Immune Complexes Requires Integrin alphaIIbbeta3 and Lyn Kinase. PLoS ONE.

[32] Hamzeh-Cognasse H., Damien P., Chabert A., Pozzetto B., Cognasse F., Garraud O. (2015). Platelets and infections-complex interactions with bacteria. Front. Immunol..

[33] Swinkels M., Rijkers M., Voorberg J., Vidarsson G., Leebeek F.W.G., Jansen A.J.G. (2018). Emerging Concepts in Immune Thrombocytopenia. Front. Immunol..

[34] Arman M., Krauel K., Tilley D.O., Weber C., Cox D., Greinacher A., Kerrigan S.W., Watson S.P. (2014). Amplification of bacteria-induced platelet activation is triggered by FcgammaRIIA, integrin alphaIIbbeta3, and platelet factor 4. Blood.

[35] Krauel K., Weber C., Brandt S., Zahringer U., Mamat U., Greinacher A., Hammerschmidt S. (2012). Platelet factor 4 binding to lipid A of Gram-negative bacteria exposes PF4/heparin-like epitopes. Blood.

[36] Krauel K., Potschke C., Weber C., Kessler W., Furll B., Ittermann T., Maier S., Hammerschmidt S., Broker B.M., Greinacher A. (2011). Platelet factor 4 binds to bacteria, [corrected] inducing antibodies cross-reacting with the major antigen in heparin-induced thrombocytopenia. Blood.

[37] Baker A.T., Boyd R.J., Sarkar D., Teijeira-Crespo A., Chan C.K., Bates E., Waraich K., Vant J., Wilson E., Truong C.D. (2021). ChAdOx1 interacts with CAR and PF4 with implications for thrombosis with thrombocytopenia syndrome. Sci. Adv..

[38] Michalik S., Siegerist F., Palankar R., Franzke K., Schindler M., Reder A., Seifert U., Cammann C., Wesche J., Steil L. (2022). Comparative analysis of ChAdOx1 nCoV-19 and Ad26.COV2.S SARS-CoV-2 vector vaccines. Haematologica.

[39] Greinacher A., Selleng K., Mayerle J., Palankar R., Wesche J., Reiche S., Aebischer A., Warkentin T.E., Muenchhoff M., Hellmuth J.C. (2021). Anti-platelet factor 4 antibodies causing VITT do not cross-react with SARS-CoV-2 spike protein. Blood.

[40] Greinacher A. (2015). CLINICAL PRACTICE. Heparin-Induced Thrombocytopenia. N. Engl. J. Med..

[41] Trepat X., Chen Z., Jacobson K. (2012). Cell migration. Compr. Physiol..

[42] Carter S.B. (1967). Haptotaxis and the mechanism of cell motility. Nature.

[43] Heckman C.A., Plummer H.K. (2013). Filopodia as sensors. Cell Signal..

[44] King S.J., Asokan S.B., Haynes E.M., Zimmerman S.P., Rotty J.D., Alb J.G., Tagliatela A., Blake D.R., Lebedeva I.P., Marston D. (2016). Lamellipodia are crucial for haptotactic sensing and response. J. Cell Sci..

[45] Bender M., Palankar R. (2021). Platelet Shape Changes during Thrombus Formation: Role of Actin-Based Protrusions. Hamostaseologie.

[46] Sandmann R., Koster S. (2016). Topographic Cues Reveal Two Distinct Spreading Mechanisms in Blood Platelets. Sci. Rep..

[47] Schurr Y., Sperr A., Volz J., Beck S., Reil L., Kusch C., Eiring P., Bryson S., Sauer M., Nieswandt B. (2019). Platelet lamellipodium formation is not required for thrombus formation and stability. Blood.

[48] Kita A., Sakurai Y., Myers D.R., Rounsevell R., Huang J.N., Seok T.J., Yu K., Wu M.C., Fletcher D.A., Lam W.A. (2011). Microenvironmental geometry guides platelet adhesion and spreading: A quantitative analysis at the single cell level. PLoS ONE.

[49] Myers D.R., Qiu Y., Fay M.E., Tennenbaum M., Chester D., Cuadrado J., Sakurai Y., Baek J., Tran R., Ciciliano J.C. (2017). Single-platelet nanomechanics measured by high-throughput cytometry. Nat. Mater..

[50] Medvedev N., Palankar R., Krauel K., Greinacher A., Delcea M. (2014). Micropatterned array to assess the interaction of single platelets with platelet factor 4-heparin-IgG complexes. Thromb. Haemost..

[51] Boylan B., Gao C., Rathore V., Gill J.C., Newman D.K., Newman P.J. (2008). Identification of FcgammaRIIa as the ITAM-bearing receptor mediating alphaIIbbeta3 outside-in integrin signaling in human platelets. Blood.

[52] Ostrowski P.P., Grinstein S., Freeman S.A. (2016). Diffusion Barriers, Mechanical Forces, and the Biophysics of Phagocytosis. Dev. Cell.

[53] Chen T., Callan-Jones A., Fedorov E., Ravasio A., Brugués A., Ong H.T., Toyama Y., Low B.C., Trepat X., Shemesh T. (2019). Large-scale curvature sensing by directional actin flow drives cellular migration mode switching. Nat. Phys..

[54] Sakurai Y., Fitch-Tewfik J.L., Qiu Y., Ahn B., Myers D.R., Tran R., Fay M.E., Ding L., Spearman P.W., Michelson A.D. (2015). Platelet geometry sensing spatially regulates alpha-granule secretion to enable matrix self-deposition. Blood.

[55] Kairdolf B.A., Smith A.M., Stokes T.H., Wang M.D., Young A.N., Nie S. (2013). Semiconductor quantum dots for bioimaging and biodiagnostic applications. Annu. Rev. Anal. Chem..

[56] Lidke D.S., Nagy P., Heintzmann R., Arndt-Jovin D.J., Post J.N., Grecco H.E., Jares-Erijman E.A., Jovin T.M. (2004). Quantum dot ligands provide new insights into erbB/HER receptor-mediated signal transduction. Nat. Biotechnol..

[57] Paknikar A.K., Eltzner B., Koster S. (2018). Direct characterization of cytoskeletal reorganization during blood platelet spreading. Prog. Biophys. Mol. Biol..

[58] Raz-Ben Aroush D., Ofer N., Abu-Shah E., Allard J., Krichevsky O., Mogilner A., Keren K. (2017). Actin Turnover in Lamellipodial Fragments. Curr. Biol..

[59] Finkenstaedt-Quinn S.A., Ge S., Haynes C.L. (2015). Cytoskeleton dynamics in drug-treated platelets. Anal. Bioanal. Chem..

[60] Jaumouille V., Farkash Y., Jaqaman K., Das R., Lowell C.A., Grinstein S. (2014). Actin cytoskeleton reorganization by Syk regulates Fcgamma receptor responsiveness by increasing its lateral mobility and clustering. Dev. Cell.

[61] Freeman S.A., Vega A., Riedl M., Collins R.F., Ostrowski P.P., Woods E.C., Bertozzi C.R., Tammi M.I., Lidke D.S., Johnson P. (2018). Transmembrane Pickets Connect Cyto- and Pericellular Skeletons Forming Barriers to Receptor Engagement. Cell.

[62] Freeman S.A., Goyette J., Furuya W., Woods E.C., Bertozzi C.R., Bergmeier W., Hinz B., van der Merwe P.A., Das R., Grinstein S. (2016). Integrins Form an Expanding Diffusional Barrier that Coordinates Phagocytosis. Cell.

[63] Lin J., Kurilova S., Scott B.L., Bosworth E., Iverson B.E., Bailey E.M., Hoppe A.D. (2016). TIRF imaging of Fc gamma receptor microclusters dynamics and signaling on macrophages during frustrated phagocytosis. BMC Immunol..

[64] Locke D., Chen H., Liu Y., Liu C., Kahn M.L. (2002). Lipid rafts orchestrate signaling by the platelet receptor glycoprotein VI. J. Biol. Chem..

