# Supplementary material for: Cytoskeleton Dependent Mobility Dynamics of FcγRIIA Facilitates Platelet Haptotaxis and Capture of Opsonized Bacteria
Source: Cells. 2022 May 11;11(10):1615. doi: 10.3390/cells11101615 (PMC9139458; doi:10.3390/cells11101615)
Supplement: Supplementary file 1 [file cells-11-01615-s001.zip › Supplementary Information for Cells Supplementary Figures S1 through S6.pdf]

Supplementary Information—Figures

Cytoskeleton Dependent Mobility Dynamics of FcγRIIA Facilitates Platelet Haptotaxis and Capture of Opsonized Bacteria

Raghavendra Palankar *, Laura Sachs, Jan Wesche and Andreas Greinacher

Affiliations: Institute for Immunology and Transfusion Medicine, University Medicine Greifswald, 17489 Greifswald, Germany
* Correspondence: author: raghavendra.palankar@med.uni-greifswald.de.

Contents:

Supplementary Figure S1. Schematics of micropatterned array functionalized with Agg-IgG used for platelet adhesion and spreading assays.

Supplementary Figure S2. Optimization of electron beam dosage for preparation of high-fidelity micropatterns.

Supplementary Figure S3. Line profiles along the fluorescent micropatterns.

Supplementary Figure S4. Characterization of quantum dots (QD) by dynamic light scattering (DLS).

Supplementary Figure S5. Confocal fluorescence microscopy of specific labeling of FcγRIIA on platelets by QD-Fab.

Supplementary Figure S6. Representative images of individual tracks and trajectories of QD-Fab bound to platelet FcγRIIA.

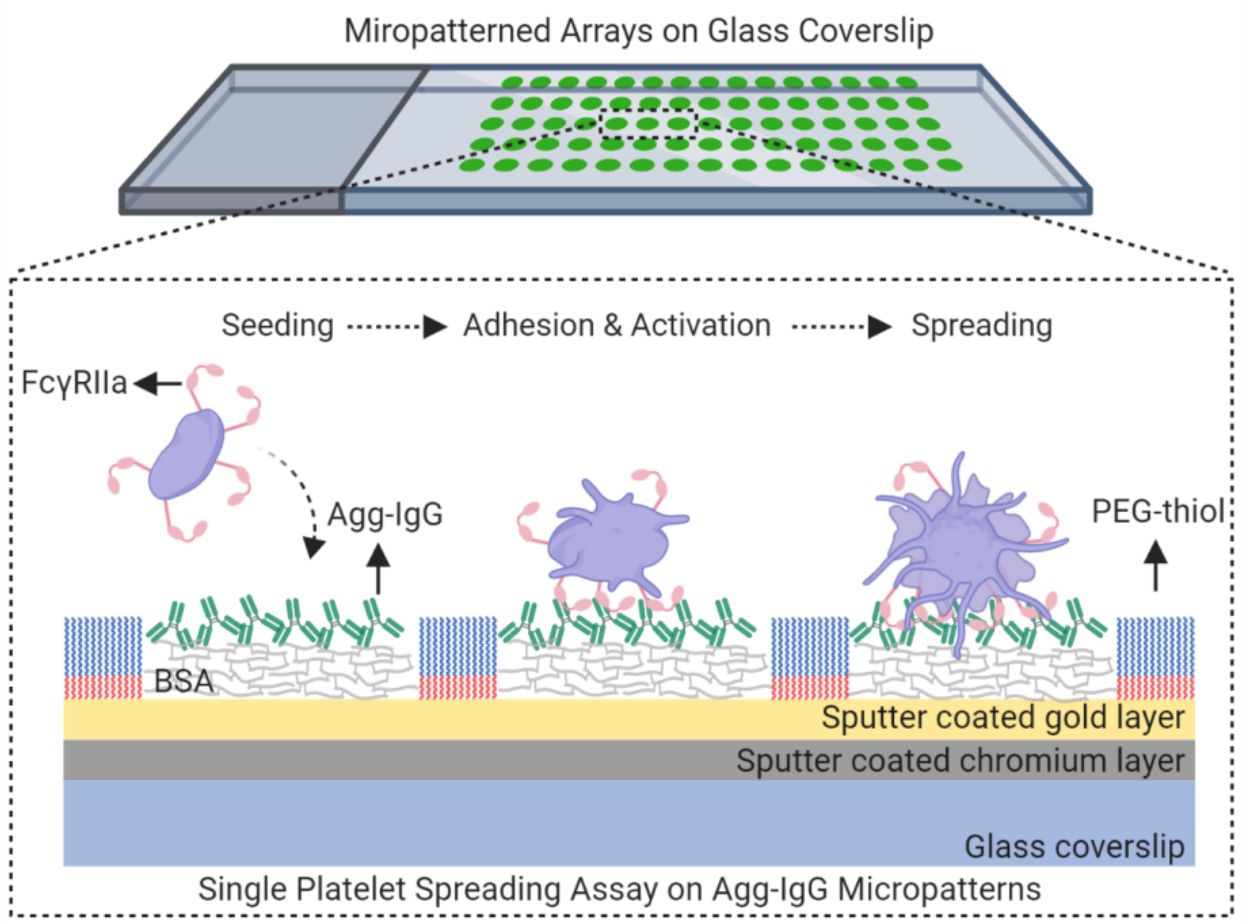

Supplementary Figure S1. Schematics of micropatterned array functionalized with Agg-IgG used for platelet adhesion and spreading assays. Micropatterned arrays were prepared using electron beam lithography on a glass coverslip sputter-coated with chromium and gold layers. The spin-coated layer of BSA was crosslinked to the gold interface utilizing an electron beam in defined micropatterns. Agg-IgG was bioconjugated to BSA, while thiolated PEG was used as a non-adhesive area that separated the micropatterns. Figure prepared with Biorender.com

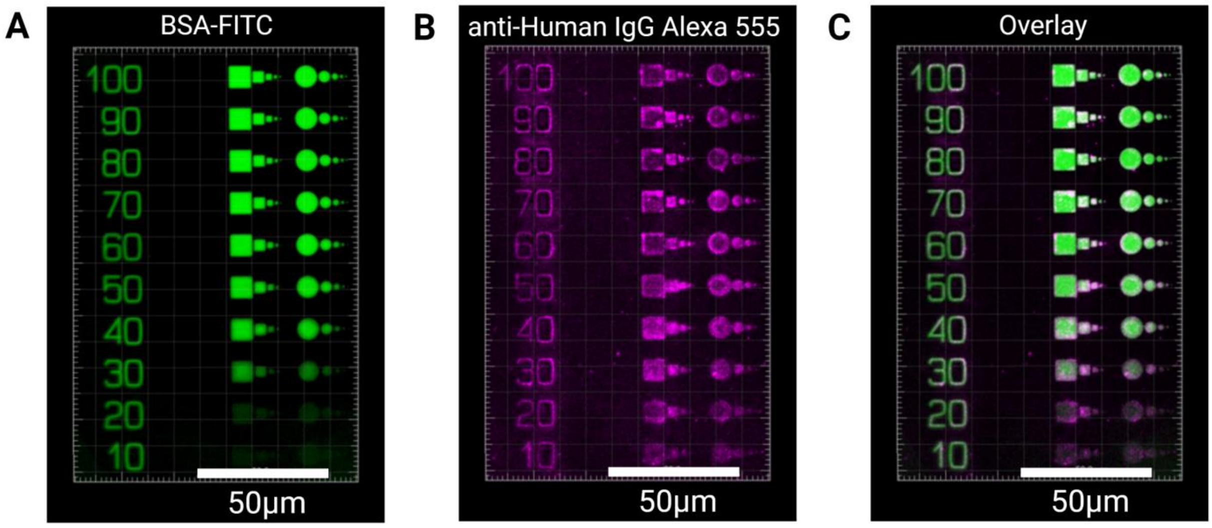

Supplementary Figure S2. Optimization of electron beam dosage for preparation of high-fidelity micropatterns. Confocal fluorescence microscopy of the impact of electron bean dosage (from 10 to 100 μC/cm²) on the fidelity and biofunctionalization of Agg-IgG (detected with anti-Human IgG Alexa 555).

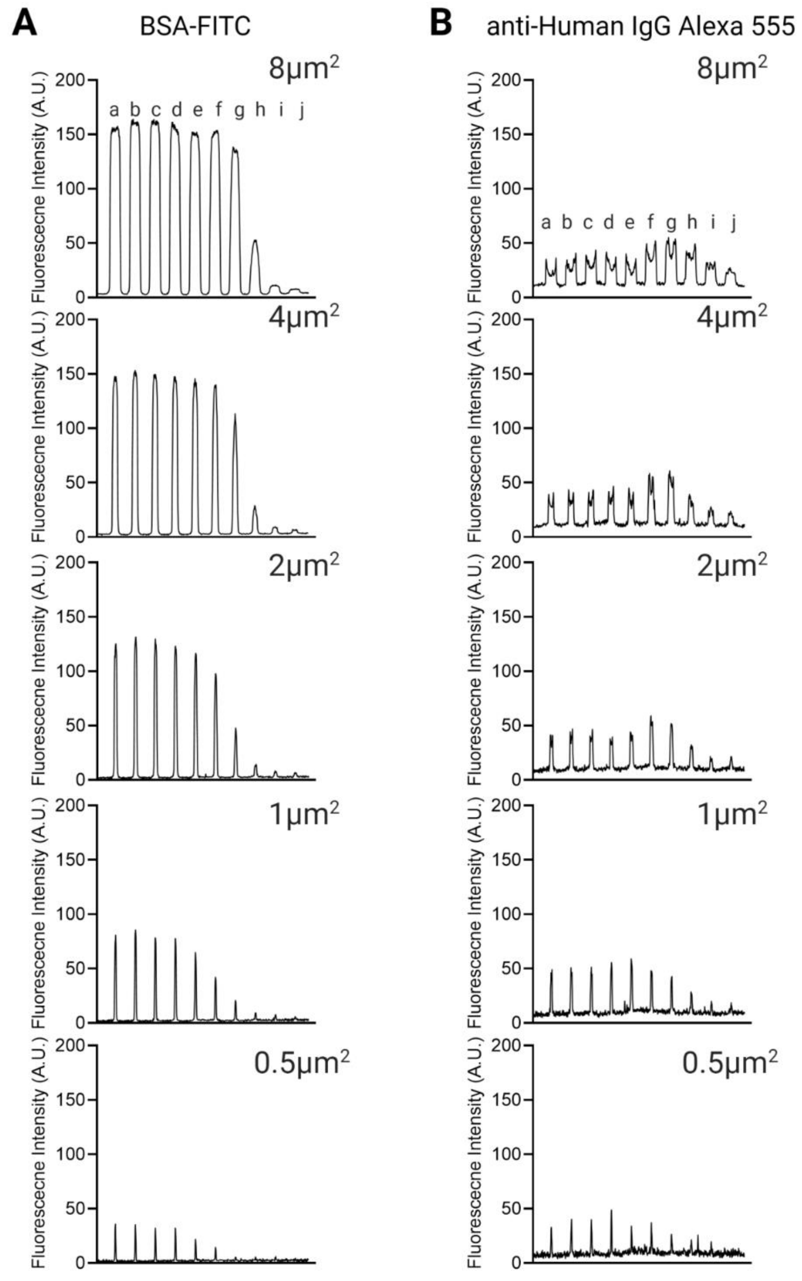

Supplementary Figure S3. Line profiles along the micropatterns prepared at different electron beam dosages (a = 100 μC/cm² a- largets dose and j = 10 μC/cm² - lowest dose) show the impact micropattern fidelity as shown by fluorescence intensities (A.U) of BSA-FITC and Agg-IgG detected by anti-human IgG Alexa 555.

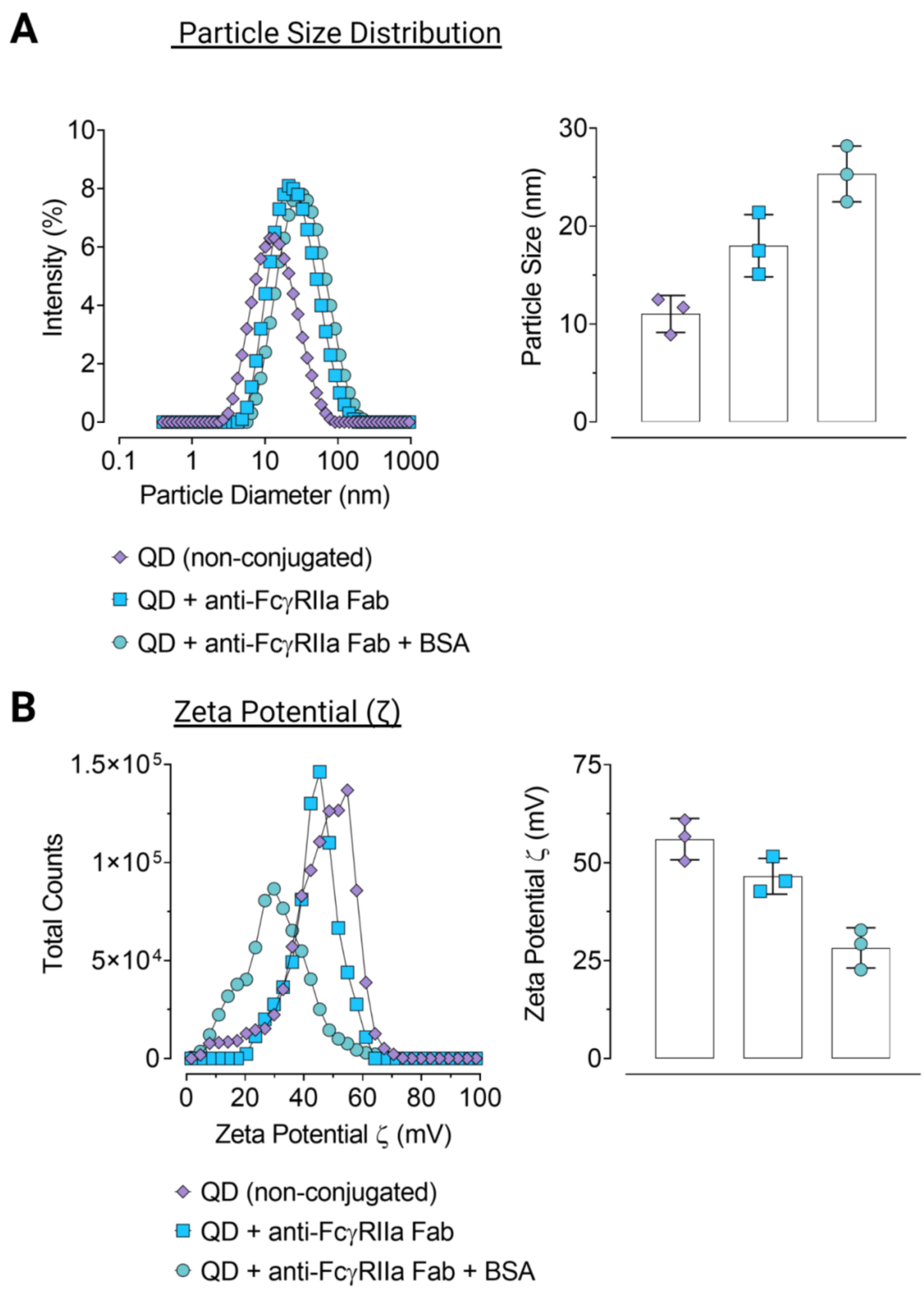

Supplementary Figure S4. Characterization of quantum dots (QD) by dynamic light scattering (DLS). Particle size (**A**) and surface zeta potential (**B**) of QD-Fab used for single-particle tracking of FcγRIIA on platelets showing changes in their physicochemical characteristics at different stages of functionalization.

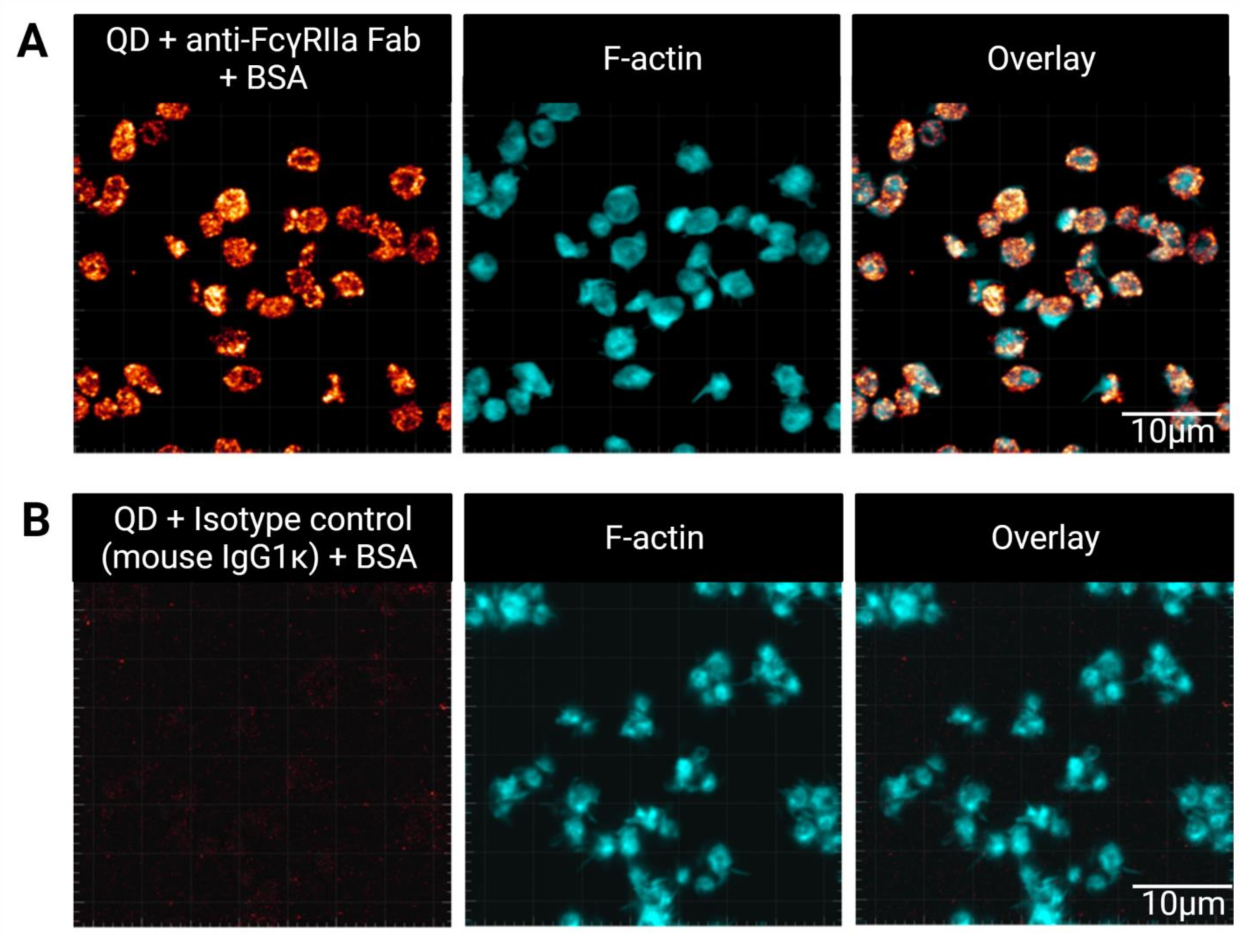

Supplementary Figure S5. Confocal fluorescence microscopy of specific labeling of FcγRIIA on platelets by QD-Fab (**A**) in comparison to the isotype control (**B**). Note: Phalloidin was used as a counter statin to visualize platelet F-actin.

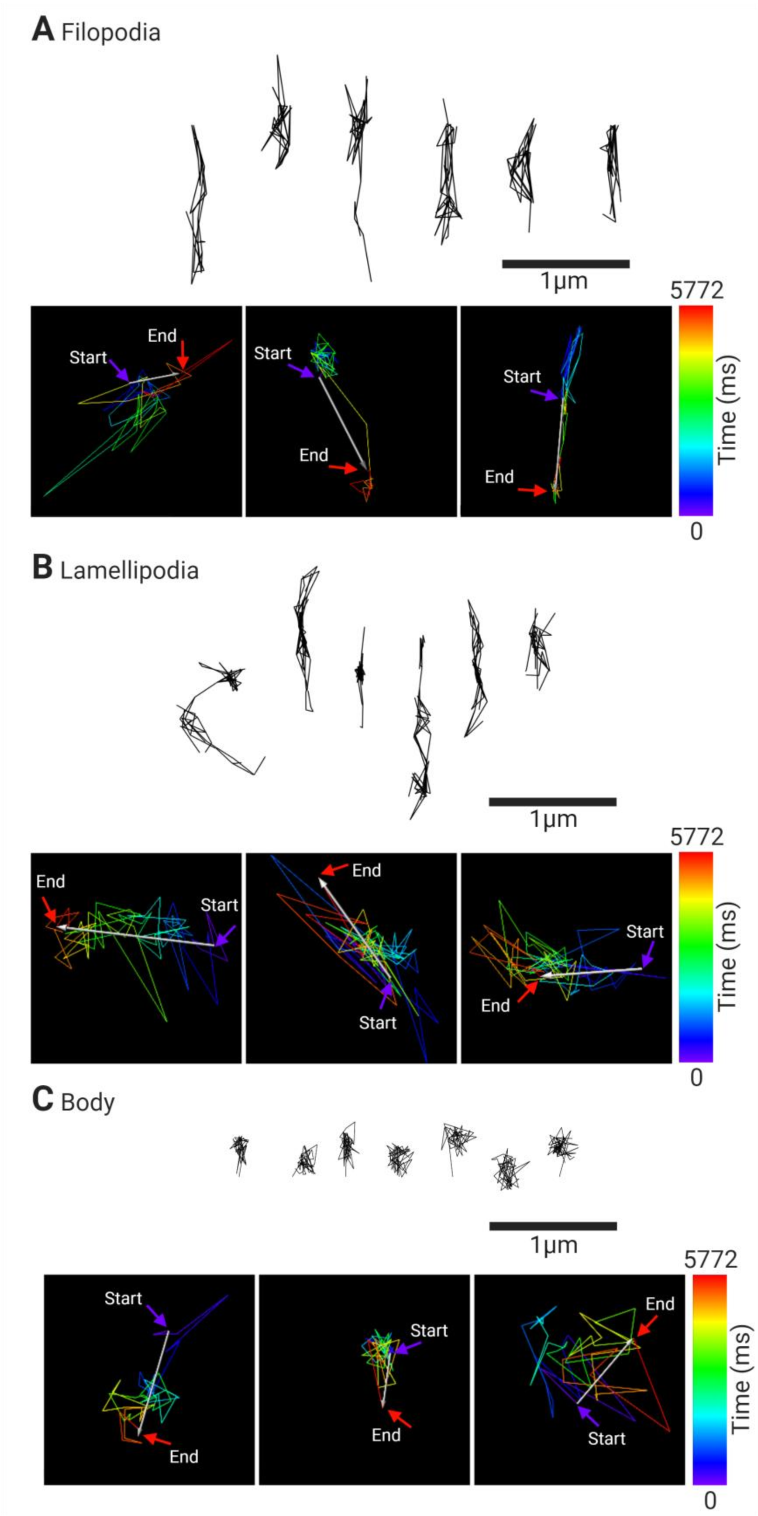

Supplementary Figure S6. Representative images of individual tracks and trajectories of QD-Fab bound to platelet FcγRIIA on filopodia (**A**), lamellipodia (**B**), and platelet body (**C**) showing differences in track lengths and QD-Fab displacement as a function of time.
