# Supplementary material for: Cytoskeleton Dependent Mobility Dynamics of FcγRIIA Facilitates Platelet Haptotaxis and Capture of Opsonized Bacteria
Source: Cells. 2022 May 11;11(10):1615. doi: 10.3390/cells11101615 (PMC9139458; doi:10.3390/cells11101615)
Supplement: Supplementary file 1 [file cells-11-01615-s001.zip › Supplementary Information for Cells Videos S1 thorugh S7.pdf]

Supplementary Information—Videos

Cytoskeleton Dependent Mobility Dynamics of FcγRIIA Facilitates Platelet Haptotaxis and Capture of Opsonized Bacteria

Raghavendra Palankar *, Laura Sachs, Jan Wesche and Andreas Greinacher

Affiliations: Institute for Immunology and Transfusion Medicine, University Medicine Greifswald, 17489 Greifswald, Germany
* Correspondence: author: raghavendra.palankar@med.uni-greifswald.de.

Contents:

Video S1. Control platelet adhesion and haptotaxis on Agg.IgG micropatterns (inter pattern pitch 1 µm) showing filopodial and lamellipodial extensions. Platelets are loaded with calcium indicator dye Fluo-4 AM to show changes in calcium release during adhesion and spreading. Playback at 5 fps.

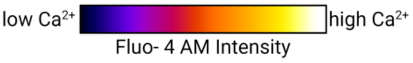

Look-up-table for intracellular Fluo-4 AM intensity

Video S2. Carier Control (DMSO 0.05%) showing platelet adhesion and haptotaxis on Agg.IgG micropatterns (inter pattern pitch 1 µm) showing filopodial and lamellipodial extensions. Platelets are loaded with calcium indicator dye Fluo-4 AM to show changes in calcium release during adhesion and spreading. Playback at 5 fps.

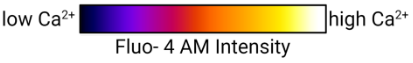

Look-up-table for intracellular Fluo-4 AM intensity

Video S3. Cytochalasin D (25 µM) treated platelets fail to adhere firmly and do not spread on Agg.IgG micropatterns (inter pattern pitch 1 µm). Platelets are loaded with calcium indicator dye Fluo-4 AM and do not show significant changes in calcium release during adhesion and spreading compared to control and DMSO-treated platelets. Playback at 5 fps.

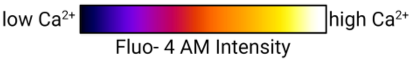

Look-up-table for intracellular Fluo-4 AM intensity

Video S4. Blebbistatin (10 µM) treated platelets adhere weakly and do not spread on Agg.IgG micropatterns (inter pattern pitch 1 µm). Platelets are loaded with calcium indicator dye Fluo-4 AM and do not show significant changes in calcium release during adhesion and spreading compared to control and DMSO-treated platelets. Playback at 5 fps.

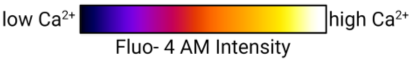

Look-up-table for intracellular Fluo-4 AM intensity

Video S5, S6, and S7. Tracking dynamics of FcγRIIA via QD-Fab on platelet filopodia, lamellipodia, and body (corresponding to Image 5B). Legends-magenta circles indicate the localization of individual QD luminescence, and yellow lines represent their computed tracks. Image interval of 78 ms, and playback is at 5 fps.
